# *Pseudomonas aeruginosa* Alters *Staphylococcus aureus* Sensitivity to Vancomycin in a Biofilm Model of Cystic Fibrosis Infection

**DOI:** 10.1128/mBio.00873-17

**Published:** 2017-07-18

**Authors:** Giulia Orazi, George A. O’Toole

**Affiliations:** Department of Microbiology and Immunology, Geisel School of Medicine at Dartmouth, Hanover, New Hampshire, USA; University of Rochester

**Keywords:** *Pseudomonas*, *Staphylococcus aureus*, antibiotic tolerance, biofilms, cystic fibrosis, polymicrobial

## Abstract

The airways of cystic fibrosis (CF) patients have thick mucus, which fosters chronic, polymicrobial infections. *Pseudomonas aeruginosa* and *Staphylococcus aureus* are two of the most prevalent respiratory pathogens in CF patients. In this study, we tested whether *P. aeruginosa* influences the susceptibility of *S. aureus* to frontline antibiotics used to treat CF lung infections. Using our *in vitro* coculture model, we observed that addition of *P. aeruginosa* supernatants to *S. aureus* biofilms grown either on epithelial cells or on plastic significantly decreased the susceptibility of *S. aureus* to vancomycin. Mutant analyses showed that 2-*n*-heptyl-4-hydroxyquinoline *N*-oxide (HQNO), a component of the *P. aeruginosa Pseudomonas* quinolone signal (PQS) system, protects *S. aureus* from the antimicrobial activity of vancomycin. Similarly, the siderophores pyoverdine and pyochelin also contribute to the ability of *P. aeruginosa* to protect *S. aureus* from vancomycin, as did growth under anoxia. Under our experimental conditions, HQNO, *P. aeruginosa* supernatant, and growth under anoxia decreased *S. aureus* growth, likely explaining why this cell wall-targeting antibiotic is less effective. *P. aeruginosa* supernatant did not confer additional protection to slow-growing *S. aureus* small colony variants. Importantly, *P. aeruginosa* supernatant protects *S. aureus* from other inhibitors of cell wall synthesis as well as protein synthesis-targeting antibiotics in an HQNO- and siderophore-dependent manner. We propose a model whereby *P. aeruginosa* causes *S. aureus* to shift to fermentative growth when these organisms are grown in coculture, leading to reduction in *S. aureus* growth and decreased susceptibility to antibiotics targeting cell wall and protein synthesis.

## IMPORTANCE

Cystic fibrosis (CF) lung infections are chronic and difficult to eradicate. *Pseudomonas aeruginosa* and *Staphylococcus aureus* are two of the most prevalent respiratory pathogens in CF patients and are associated with poor patient outcomes. Both organisms adopt a biofilm mode of growth, which contributes to high tolerance to antibiotic treatment and the recalcitrant nature of these infections. Here, we show that *P. aeruginosa* exoproducts decrease the sensitivity of *S. aureus* biofilm and planktonic populations to vancomycin, a frontline antibiotic used to treat methicillin-resistant *S. aureus* in CF patients. *P. aeruginosa* also protects *S. aureus* from other cell wall-active antibiotics as well as various classes of protein synthesis inhibitors. Thus, interspecies interactions can have dramatic and unexpected consequences on antibiotic sensitivity. This study underscores the potential impact of interspecies interactions on antibiotic efficacy in the context of complex, polymicrobial infections.

## INTRODUCTION

Cystic fibrosis (CF) is caused by mutations in the cystic fibrosis transmembrane conductance regulator (CFTR) gene. Although CF is a systemic disease, long-term lung infections are primarily responsible for poor patient outcomes (1). Despite routine administration of antibiotics, these infections are often highly resilient and resistant to treatment (2–4). Culture-independent studies have revealed that infections in the airways of CF patients are polymicrobial and complex (2–8); nonetheless, *Pseudomonas aeruginosa* and *Staphylococcus aureus* remain two of the most prevalent respiratory pathogens detected in CF patients (9). *S. aureus* is the most prevalent pathogen in younger patients with CF, while *P. aeruginosa* is highly prevalent in adult patients (9, 10). The presence of both *P. aeruginosa* and *S. aureus* is associated with decreased lung function, as measured by forced expiratory volume in 1 s (FEV_1_), and poor patient outcomes (11–14).

Interactions between *P. aeruginosa* and *S. aureus* have been the focus of several studies. Notably, it has been shown that *P. aeruginosa* negatively impacts *S. aureus* by producing 2-*n*-heptyl-4-hydroxyquinoline *N*-oxide (HQNO), an inhibitor of the electron transport chain (ETC) of *S. aureus*. HQNO can cause an increase in *S. aureus* biofilm formation at least under one condition (15, 16), and prolonged exposure to this compound leads to selection of *S. aureus* small-colony variants (SCVs) (17). Previously, HQNO has been shown to decrease the sensitivity of *S. aureus* to various aminoglycoside antibiotics, including streptomycin, dihydrostreptomycin, and tobramycin (17, 18). Additionally, HQNO and *P. aeruginosa*-produced siderophores have been shown to shift *S. aureus* to a fermentative mode of growth, eventually leading to reduced *S. aureus* viability (19–22). Finally, both *P. aeruginosa* and *S. aureus* form biofilms, which dramatically alters the expected antibiotic tolerance profiles for these organisms (23). *P. aeruginosa* forms biofilms within the CF lung, enabling persistence and recalcitrance to treatment (24–27).

In this study, we explored the effects of a dual-species interaction on the antibiotic tolerance of one microbial species in the context of CF lung infections involving bacterial biofilms. Specifically, we tested whether *P. aeruginosa* influences the susceptibility of *S. aureus* to vancomycin, a frontline antibiotic used to treat methicillin-resistant *S. aureus* (MRSA) in CF patients; approximately 25% of CF patients are culture positive for MRSA (9). We discovered that *P. aeruginosa* decreases the susceptibility of *S. aureus* biofilms to vancomycin, as well as to other cell wall synthesis inhibitors and protein synthesis inhibitors. We propose a model whereby *P. aeruginosa* exoproducts cause *S. aureus* to undergo a metabolic shift, leading to reduced growth and decreased susceptibility to a range of clinically relevant antibiotics.

## RESULTS

### *P. aeruginosa* supernatant protects *S. aureus* from vancomycin on plastic.

Previous work from our lab found that *S. aureus* 8325-4 downregulates penicillin-binding protein 4 (Pbp4) in the presence of *P. aeruginosa* when grown on plastic (22). Pbp4 has transpeptidase and carboxypeptidase activities and catalyzes the final step in peptidoglycan synthesis (28). Loss of the *pbp4* gene results in increased tolerance to vancomycin (29) and, conversely, decreased tolerance to β-lactam antibiotics (30). Thus, we hypothesized that exposure of *S. aureus* to *P. aeruginosa* might alter the susceptibility of *S. aureus* to vancomycin.

**FIG 1:**
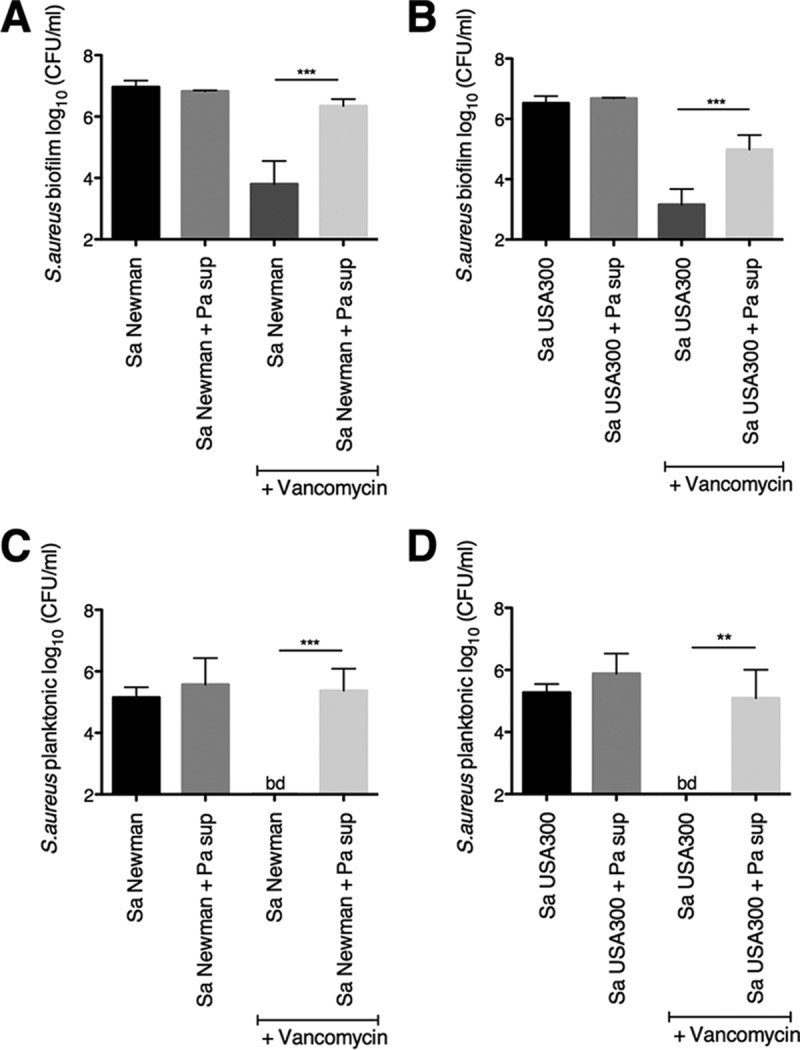
*P. aeruginosa* protects *S. aureus* biofilm and planktonic populations from vancomycin. (A to D) Biofilm disruption assays on plastic were performed with *S. aureus* (Sa) Newman (A) and USA300 (B), *P. aeruginosa* PA14 supernatant (Pa sup), and vancomycin at 50 μg/ml. *S. aureus* biofilm (A and B) and planktonic (C and D) CFU were determined. Data in panels A and C and B and D were from the same experiments. Each column displays the average from at least three biological replicates, each with three technical replicates. Error bars indicate standard deviation (SD). **, *P* < 0.01, and ***, *P* < 0.001, by ordinary one-way analysis of variance (ANOVA) and Tukey’s multiple comparisons posttest. bd, below detection.

To test this hypothesis, we selected a methicillin-sensitive *S. aureus* strain (Newman), an MRSA strain (USA300), and *P. aeruginosa* PA14 for our initial experiments. We previously showed that in our coculture system, *S. aureus* biofilm cell viability dramatically decreased after 10 to 16 h of coincubation with *P. aeruginosa* when these microbes are cocultured on CF-derived bronchial epithelial (CFBE) cells or plastic (22). In contrast, exposure of *S. aureus* to *P. aeruginosa* culture supernatant for 24 h did not alter *S. aureus* biofilm cell viability on plastic (Fig. 1A, left 2 bars). Therefore, to avoid *P. aeruginosa*-mediated, late-stage killing of *S. aureus*, we used *P. aeruginosa* supernatant to examine whether *S. aureus* vancomycin sensitivity is altered by the presence of *P. aeruginosa*-secreted products. In this assay, we first allowed *S. aureus* cells to attach for 1 h, and then the planktonic cells were removed and the attached cells washed with fresh medium and incubated for an additional 5 h to allow biofilm formation. Afterwards, the biofilm fraction was exposed to *P. aeruginosa* supernatant and/or vancomycin for 24 h. We refer to this method throughout as the “biofilm disruption assay,” because we first allow the biofilm to form and then assess the impact of different treatments on disrupting biofilm cell viability. We feel that such an assay more accurately reflects an infection-like condition wherein the microbial community is already established prior to the application of any treatment.

Incubation of *S. aureus* Newman with *P. aeruginosa* supernatant on plastic significantly decreased the sensitivity of *S. aureus* Newman biofilms to vancomycin, resulting in a 2-log increase in cell viability of *S. aureus* (Fig. 1A). Furthermore, the wild-type strain of *S. aureus* Newman and its Δ*pbp4* mutant derivative exhibited the same sensitivity to vancomycin in the absence and presence of *P. aeruginosa* supernatant, indicating that this observed decrease in sensitivity was not due to the absence of Pbp4 (Fig. 1A; see Fig. S1 in the supplemental material). Furthermore, *P. aeruginosa* supernatant protects *S. aureus* USA300 biofilms from vancomycin to a similar extent as *S. aureus* Newman (Fig. 1B).

10.1128/mBio.00873-17.2 FIG S1  The absence of Pbp4 is not responsible for decreased *S. aureus* sensitivity to vancomycin. Biofilm disruption assays on plastic were performed with the *S. aureus* (Sa) Newman *pbp4* deletion mutant (Δ*pbp4*), *P. aeruginosa* PA14 supernatant (Pa sup), and vancomycin at 50 μg/ml. Each column displays the average from at least three biological replicates, each with three technical replicates. Error bars indicate SD. ns, not significant; *, *P* < 0.05 by ordinary one-way ANOVA and Tukey’s multiple comparisons posttest. Download FIG S1, TIF file, 2.6 MB. Copyright © 2017 Orazi and O’Toole. 2017 Orazi and O’Toole This content is distributed under the terms of the Creative Commons Attribution 4.0 International license.

We also observed that *P. aeruginosa* supernatant protects *S. aureus* Newman and USA300 planktonic populations from vancomycin to levels of viability not significantly different from those of the untreated control, despite the high concentration of vancomycin used in the experiment (50 μg/ml [Fig. 1C and D]). Treatment of *S. aureus* Newman or USA300 planktonic populations with 50 μg/ml of vancomycin in the absence of *P. aeruginosa* supernatant resulted in a reduction of planktonic cell viability to below the detection level of this assay (~200 CFU/ml). Importantly, as was observed for the biofilm-grown bacteria, *P. aeruginosa* supernatant did not impact the cell viability of planktonic *S. aureus* Newman or USA300 in the absence of vancomycin (Fig. 1C and D). Furthermore, a comparison of the data from biofilm-grown and planktonic cells highlights the biofilm antibiotic tolerance of both strains versus vancomycin (Fig. 1, compare panels A and C and panels B and D).

We tested whether *P. aeruginosa* supernatant inactivates vancomycin and thus renders the drug ineffective against *S. aureus*, thereby explaining the observed increase in biofilm tolerance in the presence of *P. aeruginosa* supernatant. To test this possibility, we performed a minimum bactericidal concentration (MBC) assay in which we preincubated vancomycin with either minimal essential medium supplemented with 2 mM l-glutamine (MEM+l-Gln) or *P. aeruginosa* supernatant (prepared in MEM+l-Gln) for 24 h. The MBC of vancomycin was then determined for *S. aureus* and *Streptococcus sanguinis*. For *S. aureus*, the MBC of vancomycin that was preincubated with MEM+l-Gln alone was 3.9 μg/ml, compared to 125 μg/ml when vancomycin was preincubated with *P. aeruginosa* supernatant and MEM+l-Gln. This result is consistent with our observations in Fig. 1. For *S. sanguinis*, the MBC of vancomycin that was preincubated with MEM+l-Gln alone was 0.98 μg/ml, compared to 1.95 μg/ml when vancomycin was preincubated with *P. aeruginosa* supernatant derived in MEM+l-Gln. *S. sanguinis* remains sensitive to vancomycin in the presence of *P. aeruginosa* supernatant, indicating that *P. aeruginosa* supernatant does not inactivate vancomycin under our tested conditions.

**FIG 2:**
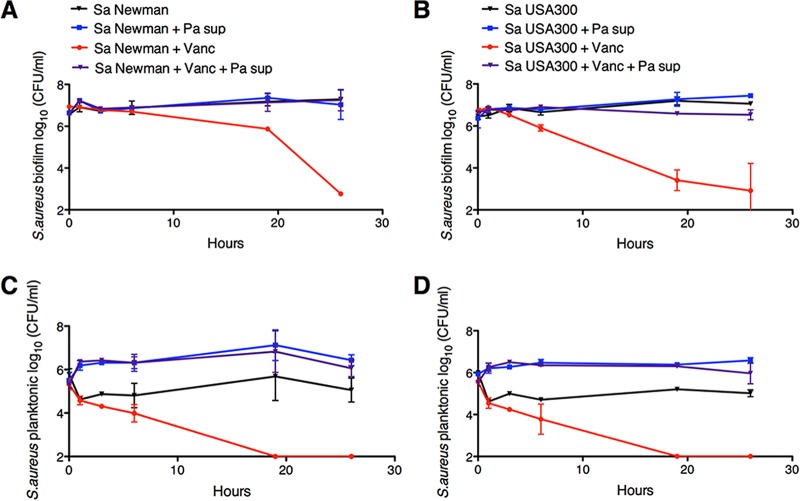
Kinetics of *S. aureus* biofilm and planktonic populations in the presence of *P. aeruginosa* supernatant and vancomycin. (A to D) Biofilm disruption assays on plastic were performed with *S. aureus* (Sa) Newman (A and C) or USA300 (B and D), *P. aeruginosa* PA14 supernatant (Pa sup), and vancomycin (Vanc) at 50 μg/ml. *S. aureus* biofilm (A and B) and planktonic (C and D) CFU were determined. Data in panels A and C and B and D were from the same experiments. Each time point displays the average from two biological replicates, each with three technical replicates. Error bars indicate SD.

We next used the coculture assay to track the kinetics of *S. aureus* tolerance to vancomycin in the presence of *P. aeruginosa* supernatant. *P. aeruginosa* supernatant promotes consistently high cell viability of biofilm-grown *S. aureus* Newman and USA300 in the presence or absence of vancomycin over the course of 26 h (Fig. 2A and B). In contrast, *S. aureus* biofilms exposed to vancomycin alone experience a steady reduction in cell viability starting at ~5 h after exposure to the antibiotic (Fig. 2A and B). *P. aeruginosa* supernatant also maintains high cell viability of *S. aureus* Newman and USA300 planktonic counterparts in the presence of vancomycin during the same time course (Fig. 2C and D).

*P. aeruginosa*-mediated protection of *S. aureus* from vancomycin is not specific to *P. aeruginosa* PA14. Another laboratory strain, *P. aeruginosa* PAO1, as well as various *P. aeruginosa* clinical isolates, is able to significantly enhance the protection of *S. aureus* Newman from vancomycin (see Fig. S2A in the supplemental material). As with *P. aeruginosa* PA14, these *P. aeruginosa* supernatants do not impact *S. aureus* Newman biofilm cell viability in the absence of vancomycin (Fig. S2B).

10.1128/mBio.00873-17.3 FIG S2  HQNO and siderophores contribute to the ability of *P. aeruginosa* to protect *S. aureus* biofilms. (A) Biofilm disruption assays on plastic were performed with *S. aureus* (Sa) Newman, supernatant (sup) from the specified *P. aeruginosa* (Pa) strain or clinical isolate, and vancomycin (Vanc) at 50 μg/ml. (B) Biofilm disruption assays on plastic were performed as described for panel A in the absence of vancomycin. (C) An illustration of the *P. aeruginosa* PQS pathway, including the protein products encoded by the relevant genes tested in the *P. aeruginosa* PA14 mutant analyses in panels D and F. (D and E) Shown is an analysis of strains with mutations in genes involved in the *P. aeruginosa* PQS biosynthetic pathway. Biofilm disruption assays on plastic were performed with *S. aureus* Newman, wild-type *P. aeruginosa* PA14, and specified deletion mutant supernatants (Pa sup), in the presence (D) or absence (E) of vancomycin (Vanc) at 50 μg/ml. (F) Biofilm disruption assays on plastic were performed with *S. aureus* USA300, supernatants from the *P. aeruginosa* PA14 wild type and the specified deletion mutants (Pa sup), and vancomycin (Vanc) at 50 μg/ml. Each column displays the average from at least two biological replicates, each with three technical replicates. Error bars indicate SD. No significant differences were found when the viability of *S. aureus* Newman exposed to each *P. aeruginosa* supernatant was compared to the viability of untreated *S. aureus* Newman (control) by ordinary one-way ANOVA and Dunnett’s multiple comparisons posttest. *, *P* < 0.05; ***, *P* < 0.001. Download FIG S2, TIF file, 19.9 MB. Copyright © 2017 Orazi and O’Toole. 2017 Orazi and O’Toole This content is distributed under the terms of the Creative Commons Attribution 4.0 International license.

### *P. aeruginosa* supernatant protects *S. aureus* from vancomycin on CFBE cells.

**FIG 3:**
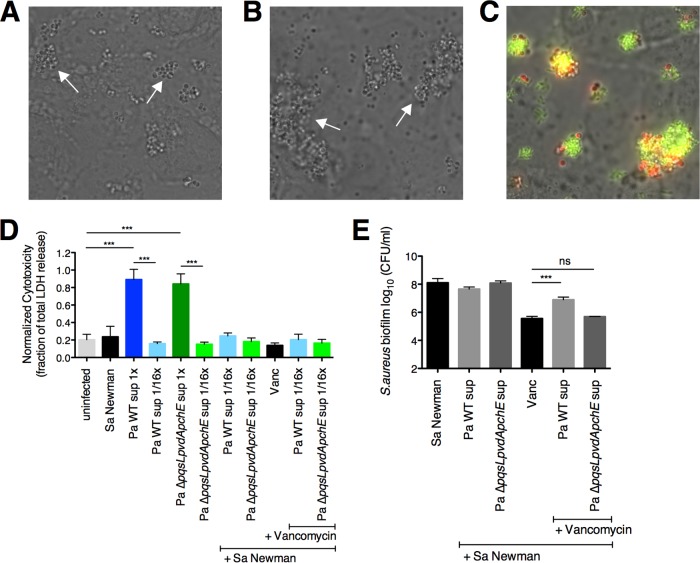
*P. aeruginosa* protects *S. aureus* biofilms from vancomycin on CFBE cells. (A and B) Representative images of *S. aureus* microcolonies on CFBE cells 6 h p.i. (A) and 21 h p.i. (B). White arrows indicate microcolonies. (C) Representative image of mixed-species microcolonies composed of *S. aureus* (red, DsRed expressing) and *P. aeruginosa* (green, green fluorescent protein [GFP] expressing) on CFBE cells 6 h p.i. (D) Cytotoxicity of *S. aureus* (Sa) Newman and/or *P. aeruginosa* PA14 supernatants (Pa sup) either undiluted (1×) or diluted 1/16× on CFBE cells. Cytotoxicity is normalized to total LDH release. (E) Biofilm disruption assays on CFBE cells were performed with *S. aureus* Newman, *P. aeruginosa* PA14 supernatant diluted 1/16×, and vancomycin (Vanc) at 50 μg/ml. Each column displays the average from at least three biological replicates, each with three technical replicates. Error bars indicate SD. ns, not significant.

Previously, we modified an established epithelial cell-*P. aeruginosa* coculture system on CFBE cells (31) to create a CFBE dual-bacterial coculture system (22). Here, we verified *S. aureus* forms biofilms on the epithelial monolayers in this system. First, we used microscopy to image *S. aureus* microcolony formation on CFBE cells (Fig. 3). We observed that *S. aureus* forms microcolonies by 6 h and continues to form biofilms for up to 21 h (Fig. 3A and B). *S. aureus* can also form mixed-species microcolonies with *P. aeruginosa* (Fig. 3C). Additionally, we performed an MBC_90_ assay on CFBE cell-grown biofilm cells to test whether the population of *S. aureus* cells attached to the airway cell monolayer exhibits high tolerance to antibiotics, a well-established feature of biofilms. The vancomycin MBC_90_ value for *S. aureus* planktonic cells was 1.95 μg/ml, compared to an MBC_90_ value of 500 μg/ml for the CFBE cell-grown biofilm fraction. Together, these data indicate that *S. aureus* forms biofilms on CFBE cells.

Next, we performed lactate dehydrogenase (LDH) cytotoxicity assays to measure the health of the CFBE cells upon exposure to *S. aureus* cells and *P. aeruginosa* supernatant under our assay conditions. Treatment with *S. aureus* Newman led to low levels of cytotoxicity similar to the MEM+l-Gln control (Fig. 3D). In contrast, undiluted *P. aeruginosa* PA14 supernatant led to high levels of cytotoxicity and disrupted the CFBE cell monolayers before the end of the experiment. When diluted 1/16×, *P. aeruginosa* PA14 supernatant showed low levels of cytotoxicity similar to the MEM+l-Gln control (Fig. 3D).

We then employed this dual-species coculture assay to test whether *P. aeruginosa* decreases *S. aureus* susceptibility to vancomycin. In this experiment, we sought to closely mirror the conditions and timing of the biofilm disruption assay on plastic described in the previous section. However, we made several modifications to the protocol used for the biofilm disruption assay on plastic to ensure that the epithelial cell monolayers remained intact throughout the experiment. We shortened the biofilm disruption assay on CFBE cells to 21 h compared to the 30-h duration for the disruption assay on plastic. Additionally, *P. aeruginosa* PA14 supernatant was diluted to preserve the integrity of the monolayers (Fig. 3D). Despite the lower concentration of supernatant used in these studies (1/16× dilution), we observed significant *P. aeruginosa-*mediated protection of *S. aureus* Newman from vancomycin on CFBE cells (Fig. 3E). Furthermore, we showed that the epithelial cytotoxicity remained low (Fig. 3D) for all the treatment conditions shown in Fig. 3E.

### HQNO and siderophores contribute to the ability of *P. aeruginosa* to protect *S. aureus* from vancomycin.

To determine which *P. aeruginosa* exoproducts are responsible for decreasing *S. aureus* sensitivity to vancomycin, we assayed strains with mutations in candidate *P. aeruginosa* PA14 genes that were previously found to be important for interactions between *P. aeruginosa* and *S. aureus* (22). The strains we tested had mutations in genes encoding *P. aeruginosa*-specific secreted products, including phenazines (*phzA-G1*/*2*), elastase (*lasB*), the master transcriptional regulators of Las and Rhl quorum sensing systems (*lasR* and *rhlR*), and several components of the *Pseudomonas* quinolone signal (PQS) quorum sensing system biosynthetic pathway (*pqsA*, *pqsH*, and *pqsL*) (see Fig. S2C in the supplemental material) and siderophore biosynthesis (*pvdA* and *pchE*). The mutant analysis was conducted by performing biofilm disruption assays on plastic using supernatants from these mutants with the addition of vancomycin.

We observed that the addition of *P. aeruginosa* PA14 deletion mutant supernatants differentially affected *S. aureus* Newman sensitivity to vancomycin. Most of the mutants tested had no impact on the ability of supernatants to confer tolerance to vancomycin (Fig. S2D). Incubation of *S. aureus* Newman with supernatant from the *P. aeruginosa* PA14 Δ*pqsA* deletion mutant resulted in a significant increase in *S. aureus* Newman sensitivity to vancomycin compared to incubation with supernatant from the wild-type *P. aeruginosa* PA14 (Fig. S2C and D). Additionally, supernatants from the *P. aeruginosa* PA14 strains carrying mutations that blocked both HQNO and siderophore production (Δ*pqsA* Δ*pvdA* Δ*pchE* and Δ*pqsL* Δ*pvdA* Δ*pchE*) resulted in a striking increase of *S. aureus* Newman sensitivity to vancomycin compared to *S. aureus* Newman treated with supernatant from the wild-type *P. aeruginosa* strain, with *S. aureus* Newman attaining levels of sensitivity similar to exposure to vancomycin alone in the absence of *P. aeruginosa* supernatant (Fig. S2D). In contrast, supernatant from the *P. aeruginosa* PA14 Δ*pqsH* deletion mutant (which eliminates PQS but not HQNO production) did not alter *S. aureus* Newman sensitivity to vancomycin compared to *S. aureus* Newman treated with supernatant from the wild-type *P. aeruginosa* strain (Fig. S2D). Taken together, it appears that the combined effect of HQNO and siderophores confers protection of *S. aureus* Newman from vancomycin. As we observed for *P. aeruginosa* PA14 wild-type supernatant, *P. aeruginosa* PA14 deletion mutant supernatants did not impact the biofilm cell viability of *S. aureus* Newman in the absence of vancomycin (Fig. S2E).

Next, we tested whether HQNO and siderophores may also play a role in decreasing *S. aureus* Newman sensitivity to vancomycin on CFBE cells. Exposure of *S. aureus* Newman to supernatant from the *P. aeruginosa* PA14 Δ*pqsL* Δ*pvdA* Δ*pchE* mutant led to a significant increase in *S. aureus* Newman susceptibility to vancomycin to levels similar to those of *S. aureus* Newman exposed to vancomycin alone (Fig. 3E). This result suggests that *P. aeruginosa*-produced HQNO and siderophores also play a role in protection of *S. aureus* Newman from vancomycin on CFBE cells.

In the case of *S. aureus* USA300, it is less clear which factors are responsible for *P. aeruginosa*-mediated protection of USA300 from vancomycin. Addition of *P. aeruginosa* PA14 supernatant from Δ*pqsA* Δ*pvdA* Δ*pchE* and Δ*pqsL* Δ*pvdA* Δ*pchE* mutants resulted in significant, but less dramatic increases in *S. aureus* USA300 susceptibility to vancomycin compared to the effect of these mutants on the susceptibility of *S. aureus* Newman (Fig. S2F). Exposure of *S. aureus* USA300 to supernatant from the *P. aeruginosa* PA14 Δ*pqsA* deletion mutant did not significantly impact *S. aureus* USA300 susceptibility to vancomycin (Fig. S2F). These findings suggest that HQNO and siderophores may be partially responsible for *P. aeruginosa*-mediated protection of *S. aureus* USA300 from vancomycin and additional factors may be involved.

**FIG 4:**
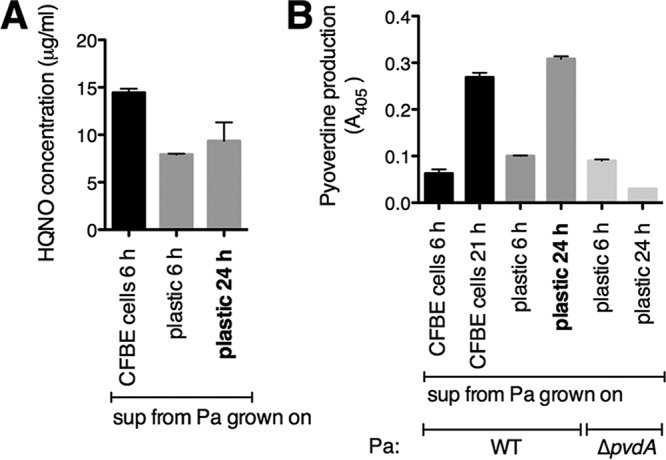
HQNO and pyoverdine quantification. (A and B) The amounts of HQNO and pyoverdine in supernatants from wild-type *P. aeruginosa* PA14 (Pa) grown either on CFBE cells or on plastic were determined. (A) Levels of HQNO were determined using a standard curve relating the HQNO concentration to *S. aureus* CFU following coculture with a *P. aeruginosa* PA14 Δ*pqsL* Δ*pvdA* Δ*pchE* deletion mutant. (B) The levels of pyoverdine were determined by measuring absorbance at 405 nm. Each column displays the average from two biological replicates, each with three technical replicates. Error bars indicate SD.

To further explore the contribution of HQNO and siderophores, we measured the levels of these exoproducts under our experimental conditions. We quantified HQNO and pyoverdine in supernatants from *P. aeruginosa* grown either on plastic or on CFBE cells in our coculture assays. To measure the levels of HQNO, we performed a functional assay in which the amount of HQNO corresponds to a *P. aeruginosa*-mediated decrease in *S. aureus* cell viability compared to a standard curve using pure, commercially available HQNO (Fig. 4A; see Fig. S3A and B in the supplemental material). A *P. aeruginosa* Δ*pqsL* mutant does not produce HQNO and thus is deficient in killing *S. aureus*; however, killing can be restored by adding exogenous HQNO. A standard curve was used to relate the HQNO concentration to *S. aureus* CFU (Fig. S3A). Using this approach, we found that by 6 h, higher levels of HQNO are present in supernatants from *P. aeruginosa* grown on CFBE cells (~15 μg/ml) compared to *P. aeruginosa* grown on plastic (~8 μg/ml HQNO [Fig. 4A]). We also measured the levels of HQNO in supernatant from *P. aeruginosa* grown on plastic for 24 h, which is the source of supernatant we use throughout the study. Under this last condition, the amount of HQNO is ~10 μg/ml, which is within the range detected in supernatant from *P. aeruginosa* grown either on CFBE cells or on plastic for 6 h (Fig. 4A).

10.1128/mBio.00873-17.4 FIG S3  Pyocyanin is not produced under our experimental conditions. (A) Shown is a standard curve relating HQNO concentration to *S. aureus* CFU following coculture with a *P. aeruginosa* (Pa) PA14 Δ*pqsL* Δ*pvdA* Δ*pchE* deletion mutant in the presence of the indicated HQNO concentrations. (B) *S. aureus* (Sa) Newman was cocultured with a *P. aeruginosa* PA14 Δ*pqsL* Δ*pvdA* Δ*pchE* deletion mutant in the presence of supernatants from wild-type *P. aeruginosa* PA14 grown either on CFBE cells or plastic. (C) Pyocyanin levels were quantified in supernatants from *P. aeruginosa* PA14 grown on either CFBE cells or plastic by measuring absorbance at 520 nm. (D and E) Biofilm disruption assays were performed with *S. aureus* Newman and the specified concentrations of pyocyanin dissolved in DMSO (D) or DMSO (E). Each column displays the average from at least two biological replicates, each with three technical replicates. Error bars indicate SD. bd, below detection; ns, not significant; ***, *P* < 0.001 by ordinary one-way ANOVA and Dunnett’s multiple comparisons posttest. Download FIG S3, TIF file, 13.2 MB. Copyright © 2017 Orazi and O’Toole. 2017 Orazi and O’Toole This content is distributed under the terms of the Creative Commons Attribution 4.0 International license.

Pyoverdine levels were measured by determining the absorbance at 405 nm, as reported previously (32). Supernatants from *P. aeruginosa* grown on plastic or on CFBE cells have low levels of pyoverdine by 6 h, similar to supernatant from the Δ*pvdA* control at 6 h (Fig. 4B). Higher levels of pyoverdine are detected in supernatants from *P. aeruginosa* grown on CFBE cells by 21 h and plastic by 24 h compared to supernatant from the Δ*pvdA* control at 24 h (Fig. 4B). These results indicate that *P. aeruginosa* produces the HQNO and pyoverdine exoproducts in our assay system, but the amounts of production differ between different growth conditions.

We also quantified the levels of the phenazine pyocyanin, another known antistaphylococcal factor produced by *P. aeruginosa*. It has been shown that pyocyanin can block the *S. aureus* ETC, leading to growth inhibition and selection for *S. aureus* SCVs (33, 34). Pyocyanin levels were determined by measuring the absorbance at 520 nm. We found that *P. aeruginosa* does not produce detectable pyocyanin under our growth conditions (Fig. S3C), perhaps explaining why this factor does not appear to play a role in *P. aeruginosa*-mediated killing of *S. aureus* in our model (22) (Fig. S2D). We verified that providing exogenous, commercially available pyocyanin, but not the vehicle control (dimethyl sulfoxide [DMSO]), does indeed decrease *S. aureus* cell viability under our conditions (Fig. S3D and E).

We have shown above that *P. aeruginosa* culture supernatant can alter *S. aureus* tolerance to vancomycin; however, we wanted to test whether coculturing *S. aureus* with *P. aeruginosa* cells could produce the same effect. Coculture of *S. aureus* Newman with wild-type *P. aeruginosa* PA14 for 21 h on CFBE cells did not lead to increased *S. aureus* tolerance to vancomycin, but instead decreased *S. aureus* cell viability (data not shown), consistent with previous findings (22). We have also shown that *P. aeruginosa* PA14 Δ*pqsL* Δ*pvdA* Δ*pchE* is unable to kill *S. aureus* (22), and here we show the same factors are required to protect *S. aureus* from vancomycin-mediated killing. We exploited the lack of killing by the Δ*pqsL* Δ*pvdA* Δ*pchE* mutant to test whether cells of these mutants are unable to protect *S. aureus* from vancomycin. Consistent with the results from supernatant experiments above, cells of the *P. aeruginosa* PA14 Δ*pqsL* Δ*pvdA* Δ*pchE* mutant are unable to protect *S. aureus* from vancomycin (data not shown).

### Exogenous HQNO protects *S. aureus* biofilms from vancomycin.

**FIG 5:**
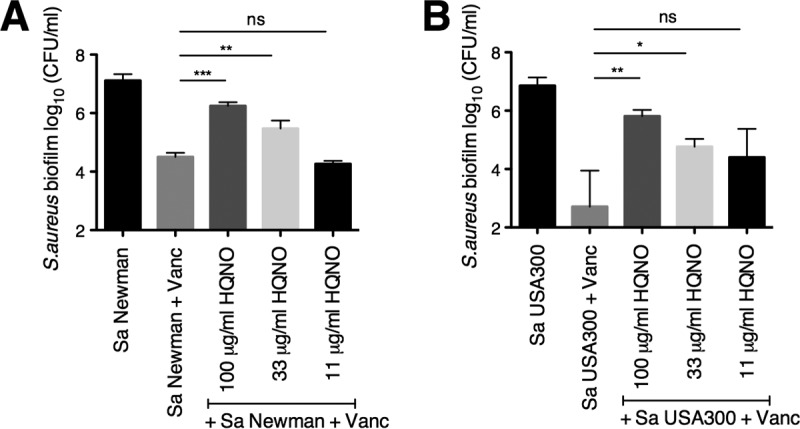
Exogenous HQNO protects *S. aureus* biofilms from vancomycin. (A and B) Biofilm disruption assays on plastic were performed with *S. aureus* (Sa) Newman (A) or USA300 (B), vancomycin (Vanc) at 50 μg/ml, and the specified concentrations of HQNO (dissolved in DMSO). Each column displays the average from at least three biological replicates, each with three technical replicates. Error bars indicate SD. ns, not significant; *, *P* < 0.05, **, *P* < 0.01, and ***, *P* < 0.001, by ordinary one-way ANOVA and Tukey’s multiple comparisons posttest.

To confirm the contribution of HQNO in altering the sensitivity of *S. aureus* to vancomycin, we conducted a biofilm disruption assay on plastic with the addition of pure HQNO (Fig. 5), with and without vancomycin. We used 100, 33, and 11 μg/ml of HQNO in our study, concentrations in the range of those measured above and produced by stationary-phase *P. aeruginosa* cultures in rich media (35, 36) (Fig. 4). We observed that exogenous HQNO protects *S. aureus* Newman and USA300 from vancomycin in an HQNO dose-dependent manner (Fig. 5A and B). The concentrations of HQNO and DMSO used in our study did not reduce *S. aureus* Newman biofilm cell viability (see Fig. S4A and B in the supplemental material). Furthermore, HQNO-mediated protection of *S. aureus* from vancomycin is not specific to *S. aureus* Newman and USA300; exogenous HQNO decreased the sensitivity to vancomycin of two out of four *S. aureus* clinical isolates tested (see Fig. S5A to D in the supplemental material). Finally, we showed that this protection was mediated specifically by HQNO because the addition of high levels of PQS, another end product of this pathway (Fig. S2C), did not protect *S. aureus* Newman biofilms from vancomycin treatment (Fig. S5E).

10.1128/mBio.00873-17.5 FIG S4  The concentrations of HQNO and DMSO used in this study do not impact the viability of *S. aureus* Newman biofilms in the absence of vancomycin, and DMSO does not alter sensitivity of *S. aureus* Newman biofilms to vancomycin. (A) Biofilm disruption assays on plastic were performed with *S. aureus* (Sa) Newman and the specified concentrations of 2-*n*-heptyl-4-hydroxyquinoline *N*-oxide (HQNO, dissolved in DMSO) and DMSO. (B) Biofilm disruption assays on plastic were performed with *S. aureus* Newman, the specified concentrations of DMSO, and vancomycin (Vanc) at 50 μg/ml. Each column displays the average from at least three biological replicates, each with three technical replicates. Error bars indicate SD. ns, not significant. Download FIG S4, TIF file, 2.1 MB. Copyright © 2017 Orazi and O’Toole. 2017 Orazi and O’Toole This content is distributed under the terms of the Creative Commons Attribution 4.0 International license.

10.1128/mBio.00873-17.6 FIG S5  HQNO protects *S. aureus* clinical isolates from vancomycin to various degrees. (A to D) Biofilm disruption assays on plastic were performed with the specified *S. aureus* (Sa) clinical isolate, 100 μg/ml of HQNO, and vancomycin at 50 μg/ml. The SMC number indicates the strain number used. (E) Biofilm disruption assays on plastic were performed with *S. aureus* Newman, 100 μg/ml of PQS, and vancomycin at 50 μg/ml. Each column displays the average from at least three biological replicates, each with three technical replicates. Error bars indicate SD. ns, not significant; *, *P* < 0.05, by ordinary one-way ANOVA and Tukey’s multiple comparisons posttest. Download FIG S5, TIF file, 14.1 MB. Copyright © 2017 Orazi and O’Toole. 2017 Orazi and O’Toole This content is distributed under the terms of the Creative Commons Attribution 4.0 International license.

### Anoxia protects *S. aureus* biofilms from vancomycin.

Our previous studies indicated that in coculture, *P. aeruginosa* interference with *S. aureus* ETC function results in *S. aureus* growing via fermentation (22). That is, growth in the presence of *P. aeruginosa* shifted *S. aureus* to anoxic-like growth conditions. Here, we sought to determine whether anoxic conditions impact *S. aureus* biofilm susceptibility to vancomycin under our specific assay conditions, and if so, whether anoxic conditions recapitulate *P. aeruginosa*-mediated protection of *S. aureus* from vancomycin.

**FIG 6:**
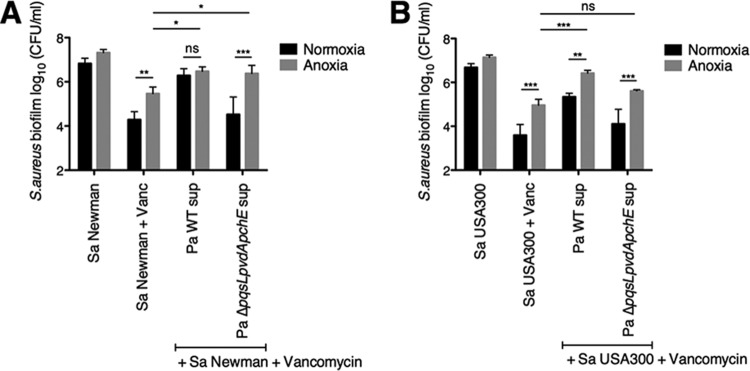
Anoxia protects *S. aureus* biofilms from vancomycin. (A and B) Biofilm disruption assays on plastic were performed with *S. aureus* (Sa) Newman (A) or USA300 (B), *P. aeruginosa* PA14 (Pa) wild-type (WT) and Δ*pqsL*Δ *pvdA* Δ*pchE* deletion mutant supernatants, and vancomycin at 50 μg/ml under either normoxic or anoxic conditions. Each column displays the average from at least three biological replicates, each with three technical replicates. Error bars indicate SD. ns, not significant; *, *P* < 0.05, **, *P* < 0.01, and ***, *P* < 0.001, by ordinary one-way ANOVA and Tukey’s multiple comparisons posttest.

Under our assay conditions for biofilms grown on a plastic surface, anoxia decreased *S. aureus* sensitivity to vancomycin (Fig. 6A and B), which is consistent with a previous finding (37). Additionally, it was reported that while the MICs of vancomycin for *S. aureus* were similar under normoxia and anoxia, the rate of *S. aureus* planktonic cell death is greater in the presence of oxygen (38, 39). Addition of supernatant from wild-type *P. aeruginosa* PA14 under anoxic conditions conferred additional protection to *S. aureus* Newman and USA300 from vancomycin compared to normoxia alone (Fig. 6A and B). Under anoxic conditions, supernatant from the *P. aeruginosa* PA14 Δ*pqsL* Δ*pvdA* Δ*pchE* mutant conferred protection to both *S. aureus* Newman and USA300, illustrating that anoxia recapitulates the activity of *P. aeruginosa* supernatant in protecting *S. aureus* biofilms from vancomycin (Fig. 6A and B).

### *P. aeruginosa* supernatant decreases *S. aureus* growth.

Our data above indicate that *P. aeruginosa* supernatant, likely via HQNO and siderophores, protects *S. aureus* biofilms from vancomycin treatment. Previous studies have observed HQNO-dependent growth inhibition of *S. aureus* (17, 40, 41). Thus, one likely mechanism for the reduced efficacy of vancomycin observed here, given its cell wall target, is slowed growth of the target bacterium.

**FIG 7:**
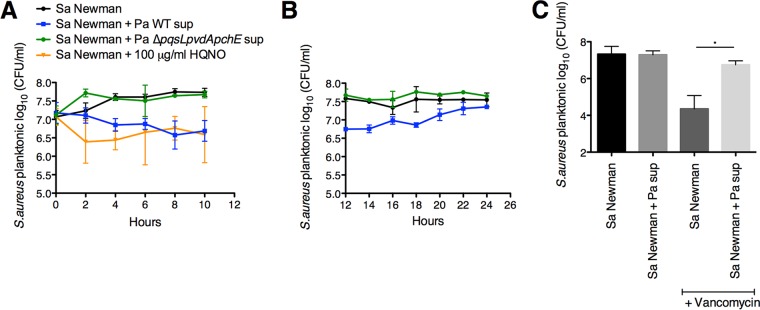
*P. aeruginosa* supernatant decreases *S. aureus* growth. (A and B) Growth curve assays of planktonic populations in shaking flasks were performed with *S. aureus* (Sa) Newman, HQNO at 100 μg/ml, and *P. aeruginosa* PA14 (Pa) wild-type (WT) and Δ*pqsL* Δ*pvdA* Δ*pchE* deletion mutant supernatants. Samples were collected either every 2 h from 0 to 10 h p.i. (A) or every 2 h from 12 h to 24 h p.i. (B). Each time point displays the average from at least three biological replicates, each with two technical replicates. Error bars indicate SD. (C) Planktonic susceptibility assays in shaking flasks were performed with *S. aureus* (Sa) Newman, *P. aeruginosa* PA14 supernatant (Pa sup), and 50 μg/ml of vancomycin. Each column displays the average from at two biological replicates, each with two technical replicates. Error bars indicate SD. *, *P* < 0.05 by ordinary one-way ANOVA and Tukey’s multiple comparisons posttest.

We tested whether exposure of *S. aureus* to exogenous HQNO or supernatants from wild-type *P. aeruginosa* PA14 or the Δ*pqsL* Δ*pvdA* Δ*pchE* mutant would result in reduced *S. aureus* growth. In this experiment, we monitored growth of *S. aureus* over the course of 10 h in shaking flask cultures with the same medium used in the coculture assays (MEM+l-Gln), alone or amended with HQNO or *P. aeruginosa* supernatant. Exposure of *S. aureus* to HQNO and *P. aeruginosa* PA14 wild-type supernatant caused a decrease in *S. aureus* growth compared to *S. aureus* grown in MEM+l-Gln alone (control [Fig. 7A]). Conversely, *S. aureus* treated with *P. aeruginosa* PA14 Δ*pqsL* Δ*pvdA* Δ*pchE* mutant supernatant resulted in a growth profile similar to that of the control (Fig. 7A); both the control and the culture amended with *P. aeruginosa* PA14 Δ*pqsL* Δ*pvdA* Δ*pchE* mutant supernatant showed an ~1-log increase in viability between 8 and 14 h. Next, we performed an extended growth curve experiment to monitor growth of *S. aureus* from 12 to 24 h in the presence or absence of *P. aeruginosa* supernatants. Starting at 16 h, there is an increase in growth of *S. aureus* exposed to *P. aeruginosa* PA14 wild-type supernatant, reaching levels similar to those of the control and the culture treated with *P. aeruginosa* PA14 Δ*pqsL* Δ*pvdA* Δ*pchE* mutant supernatant by 24 h (Fig. 7B). Thus, while supernatants and HQNO slow the growth of *S. aureus* in the short term, these treatments do not cause long-term loss of viability, an observation consistent with our conclusion that protection of *S. aureus* from vancomycin treatment in coculture is likely due to the slower growth of this Gram-positive organism.

As a control, we verified that *P. aeruginosa* supernatant protects *S. aureus* from vancomycin when grown in shaking flasks (Fig. 7C), the assay conditions used to monitor growth in Fig. 7A and B. Overall, these data indicate that *P. aeruginosa*-derived HQNO and siderophores can reduce or eliminate *S. aureus* growth in the minimal medium used in these studies.

### Exposure to *P. aeruginosa* supernatant does not select for SCVs under our experimental conditions.

*S. aureus* SCVs arise due to mutations in the ETC, resulting in high tolerance to antibiotic treatment (42–45). Previous studies reported that exposure of *S. aureus* to *P. aeruginosa* or pure HQNO can select for *S. aureus* SCVs (15, 17, 34). To determine whether SCVs are playing a role in promoting *S. aureus* biofilm cell viability in our model, we sought to enumerate any SCVs that may arise under our experimental conditions. We conducted biofilm disruption assays on plastic for up to 5 days in which *S. aureus* Newman was exposed to either MEM+l-Gln alone (control), or supernatants derived from wild-type *P. aeruginosa* PA14 or the Δ*pqsL* Δ*pvdA* Δ*pchE* mutant. We did not observe SCVs under any of the conditions tested.

### *P. aeruginosa* supernatant does not further enhance the tolerance of a highly resistant *S. aureus* small colony variant to vancomycin.

**FIG 8:**
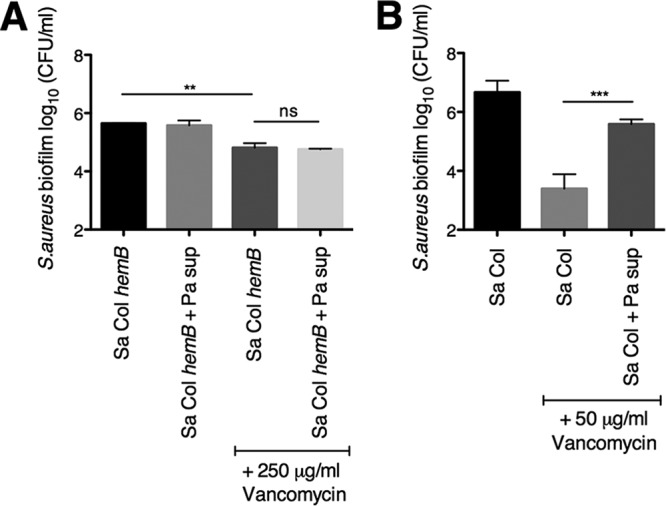
*S. aureus* small-colony variant biofilms are tolerant to vancomycin independent of *P. aeruginosa* supernatant. (A and B) Biofilm disruption assays on plastic were performed with *S. aureus* (Sa) Col, *P. aeruginosa* PA14 supernatant (Pa sup), and vancomycin. (A) The *S. aureus* Col *hemB* mutant was exposed to 250 μg/ml of vancomycin. (B) The *S. aureus* Col parental strain was exposed to 50 μg/ml of vancomycin. Each column displays the average from at least three biological replicates, each with three technical replicates. Error bars indicate SD. ns, not significant; **, P < 0.01, and ***, *P* < 0.001, by ordinary one-way ANOVA and Tukey’s multiple comparisons posttest.

To further probe whether SCVs might contribute to the *P. aeruginosa*-mediated protection of *S. aureus* from vancomycin, we used a previously described *S. aureus* SCV, generated by mutating a key gene for heme biosynthesis, *hemB* (44). An *S. aureus* Col *hemB* SCV variant has a defective ETC and the classical small colony phenotype (44, 46–49). The *S. aureus* Col *hemB* mutant is highly tolerant to vancomycin (Fig. 8A), which is consistent with previous findings that SCVs are tolerant to other cell wall-active antibiotics (42, 44). We observed that *P. aeruginosa* PA14 supernatant did not further enhance the tolerance of the *S. aureus* Col *hemB* mutant to vancomycin (Fig. 8A), which is consistent with the mechanism by which ETC inhibition confers tolerance to this antibiotic. In contrast to the *S. aureus* Col *hemB* mutant, the Col parental strain is susceptible to vancomycin to levels similar to those of *S. aureus* Newman, and likewise, *P. aeruginosa* supernatant protected the *S. aureus* Col parental strain from vancomycin (Fig. 8B).

### *P. aeruginosa* supernatant reduces *S. aureus* cell wall thickness.

A commonly observed feature of vancomycin-resistant strains of *S. aureus* is an increase in cell wall thickness (50, 51). *S. aureus* cells grown anaerobically also exhibit an increase in cell wall thickness (52). We examined whether exposure of biofilm-grown *S. aureus* Newman to *P. aeruginosa* PA14 supernatant causes *S. aureus* cell wall thickening, which may explain the observed decreased susceptibility to vancomycin of *S. aureus* in the presence of *P. aeruginosa*. Surprisingly, we found that *S. aureus* Newman cells exposed to MEM+l-Gln alone (control) for 24 h had very thick cell walls (average of 70 nm [see Fig. S6A and D in the supplemental material]). In contrast, *S. aureus* Newman biofilm cells that were exposed to *P. aeruginosa* supernatant for 24 h had cell walls significantly less thick relative to the control (average of 31 nm [Fig. S6B and D]), values that were comparable to those reported for *S. aureus* cells grown in rich medium (50, 51). Next, we tested whether HQNO could be mediating the observed decrease in *S. aureus* cell wall thickness upon exposure to *P. aeruginosa* supernatant. Indeed, *S. aureus* Newman biofilm cells treated with exogenous HQNO had less thick cell walls relative to the control (average of 28 nm [Fig. S6C and E]), which recapitulated the effect of *P. aeruginosa* PA14 supernatant. These results indicate that thickening of the *S. aureus* Newman cell wall is not the basis for increased vancomycin tolerance when exposed to *P. aeruginosa* PA14 supernatant.

10.1128/mBio.00873-17.7 FIG S6  *P. aeruginosa* supernatant decreases the cell wall thickness of biofilm-grown *S. aureus*. (A to C) Representative transmission electron microscopy (TEM) images of *S. aureus* Newman grown in MEM + l-Gln alone (control [A]) or in the presence of either *P. aeruginosa* PA14 supernatant (B) or 100 μg/ml of HQNO (C). (D and E) Cell wall thickness was measured for *S. aureus* (Sa) Newman cells grown as biofilms incubated with or without *P. aeruginosa* PA14 supernatant (Pa sup) for 24 h (D) or with or without 100 μg/ml of HQNO (dissolved in DMSO) for 24 h (E). Each column displays the average of two biological replicates, each with two technical replicates. Error bars indicate SD. *, *P* < 0.05, and ***, *P* < 0.001, by two-tailed unpaired *t* test. Download FIG S6, TIF file, 3.3 MB. Copyright © 2017 Orazi and O’Toole. 2017 Orazi and O’Toole This content is distributed under the terms of the Creative Commons Attribution 4.0 International license.

### *P. aeruginosa* supernatant protects *S. aureus* from other antibiotics.

The data presented here show that *P. aeruginosa* supernatant can protect *S. aureus* biofilms from the antimicrobial effect of vancomycin. We wanted to ask how broadly this protection extended to other antibiotics. We found that this protection phenotype is not specific to vancomycin; *P. aeruginosa* PA14 supernatant protects *S. aureus* Newman from another cell wall-active antibiotic—oxacillin (see Fig. S7A in the supplemental material). In contrast, *P. aeruginosa* supernatant does not protect *S. aureus* Newman or USA300 biofilms from the antibiotic daptomycin (Fig. S7B and C). Unlike vancomycin and oxacillin, daptomycin does not target the cell wall of *S. aureus*.

10.1128/mBio.00873-17.8 FIG S7  Testing *P. aeruginosa-*mediated protection of *S. aureus* biofilms from other antibiotics. (A) Biofilm disruption assays on plastic were performed with *S. aureus* (Sa) Newman, *P. aeruginosa* PA14 supernatant (Pa sup), and the specified concentration of oxacillin. (B and C) Biofilm disruption assays on plastic were performed with *S. aureus* Newman (B) or USA300 (C), *P. aeruginosa* PA14 supernatant, and the specified concentrations of daptomycin. Each column displays the average from at least three biological replicates, each with three technical replicates. Error bars indicate SD. ns, not significant; *, *P* < 0.05 by ordinary one-way ANOVA and Tukey’s multiple comparisons posttest. Download FIG S7, TIF file, 1.8 MB. Copyright © 2017 Orazi and O’Toole. 2017 Orazi and O’Toole This content is distributed under the terms of the Creative Commons Attribution 4.0 International license.

To more thoroughly test whether *P. aeruginosa* supernatant can protect *S. aureus* from other antibiotic classes, we screened Biolog Phenotype MicroArray panels containing 240 antibiotics for a supernatant-mediated protection phenotype. For each panel, *S. aureus* Newman was exposed to either MEM+l-Gln alone (control) or *P. aeruginosa* PA14 wild-type supernatant. Interestingly, it appears that *P. aeruginosa* supernatant can protect *S. aureus* from a wide range of antibiotics, many of which fall into two broad categories: cell wall synthesis inhibitors and protein synthesis inhibitors (see Table S1 in the supplemental material). Specifically, these antibiotics include multiple representatives from the β-lactam, glycopeptide, aminoglycoside, macrolide, and tetracycline classes. Importantly, we confirmed our previous findings that *P. aeruginosa* supernatant protects *S. aureus* from vancomycin and oxacillin (Table S1; Fig. S7). We also observed that *P. aeruginosa* supernatant reduces *S. aureus* sensitivity to the nucleic acid inhibitors rifampin and novobiocin, as well as numerous other chemicals (Table S1).

10.1128/mBio.00873-17.9 TABLE S1  *P. aeruginosa* supernatant protects *S. aureus* from several classes of antibiotics. Download TABLE S1, PDF file, 0.1 MB. Copyright © 2017 Orazi and O’Toole. 2017 Orazi and O’Toole This content is distributed under the terms of the Creative Commons Attribution 4.0 International license.

Furthermore, we tested whether HQNO and siderophores are required for protection of *S. aureus* from a subset of antibiotics in the Phenotype MicroArray panels. *S. aureus* Newman was added to panel 12 and exposed to either MEM+l-Gln alone (control) or *P. aeruginosa* PA14 wild-type or Δ*pqsL* Δ*pvdA* Δ*pchE* mutant supernatants. We found that HQNO and siderophores contribute to the ability of *P. aeruginosa* to protect *S. aureus* from cell wall-targeting antibiotics (β-lactam class), protein synthesis inhibitors (aminoglycoside, macrolide, and tetracycline classes), and nucleic acid inhibitors (rifampin and novobiocin) (Table S1).

## DISCUSSION

In this study, we have shown that interspecies bacterial interactions alter antibiotic tolerance in unpredictable ways. Specifically, we found that *P. aeruginosa* protects biofilm and planktonic populations of *S. aureus* from vancomycin, a frontline drug to treat MRSA in CF patients and in other infections. Furthermore, we observed that *P. aeruginosa*-mediated protection of *S. aureus* from vancomycin occurs with multiple *P. aeruginosa* and *S. aureus* strains, as well as clinical isolates.

We have shown that the *P. aeruginosa* exoproducts HQNO and siderophores contribute to protection of *S. aureus* from vancomycin. We previously demonstrated that both factors are required to shift *S. aureus* from respiration to fermentation, thus slowing the growth of the bacterium (22). To test whether inhibition of electron transport resulted in increased tolerance to vancomycin, we exposed *S. aureus* to anoxic, fermentative conditions to inhibit respiration in a different way. Anoxia recapitulated the effect of *P. aeruginosa* supernatant. Overall, we propose that the enhanced tolerance of the biofilm is mediated, at least in part, by the reduced growth of *S. aureus* when this microbe is grown in the presence of *P. aeruginosa* exoproducts. Our growth assays support this hypothesis. In contrast, our data do not support a role for SCVs in *P. aeruginosa*-mediated protection of *S. aureus* from vancomycin in our model system. Consistent with our model, it has been shown that anoxia also slows *S. aureus* growth in minimal medium (53). Additionally, it has been reported that *S. aureus* also exhibits slow growth in sputum from CF patients, raising the possibility of increased *S. aureus* tolerance to vancomycin *in vivo* (54).

We observed that *P. aeruginosa* supernatant protects *S. aureus* from antibiotics with two broad mechanisms of action: cell wall synthesis inhibitors and protein synthesis inhibitors. Thus, the interaction we observe here could have a general effect on antimicrobial therapy in the context of polymicrobial infections. Furthermore, we showed that HQNO and siderophores are required for protection from representatives from both cell wall-targeting antibiotics and protein synthesis inhibitors. We suggest above that slowed growth and/or the shift to fermentative growth of *S. aureus* by *P. aeruginosa* exoproducts confers resistance to vancomycin, oxacillin, and other cell wall-active antibiotics. Similarly, we propose a model wherein HQNO- and siderophore-mediated inhibition of the ETC disrupts the electrochemical gradient and thus prevents cell entry of protein synthesis inhibitors that require protein motive force, including aminoglycosides and tetracyclines (55). Furthermore, the mechanism by which *P. aeruginosa* alters *S. aureus* antibiotic tolerance here may extend to other polymicrobial interactions. For example, several *P. aeruginosa*-derived phenazines repress *Candida albicans* respiratory metabolism and drive this fungus toward fermentative metabolism and the production of ethanol (56–58).

We also tested whether *P. aeruginosa* supernatant alters the sensitivity of *S. aureus* to daptomycin, which acts via a different mechanism. Daptomycin inserts into the cell membrane, leading to membrane depolarization, a loss of membrane potential, and subsequent death of the bacterial cell. *P. aeruginosa* supernatant does not protect *S. aureus* from daptomycin, further suggesting that *P. aeruginosa*-mediated protection of *S. aureus* is dependent on the antibiotic’s mode of action. Moreover, in our Biolog Phenotype MicroArray screen, we observed that *P. aeruginosa* supernatant did not protect *S. aureus* from nucleic acid inhibitors apart from rifampin and novobiocin.

*P. aeruginosa* produces several known antistaphylococcal factors: HQNO, siderophores, pyocyanin, and rhamnolipids (18, 22, 33, 59, 60). Pyocyanin does not seem to be involved in *P. aeruginosa-S. aureus* interactions here because it is not produced in appreciable amounts by *P. aeruginosa* under our experimental conditions (Fig. S3C). In our system, HQNO and siderophores are involved in multiple *P. aeruginosa-S. aureus* interactions: *P. aeruginosa-*mediated killing of *S. aureus* (22) and *P. aeruginosa-*mediated protection of *S. aureus* from antibiotics. *P. aeruginosa*-mediated killing of *S. aureus* occurs after 10 to 16 h of coculture, whereas *P. aeruginosa*-mediated tolerance of *S. aureus* to vancomycin can occur after as early as 6 h, indicating the possibility that these interactions could be occurring sequentially. Additionally, *S. aureus* cells could be experiencing these *P. aeruginosa* exoproducts at a distance, resulting in a gradient of *P. aeruginosa-*mediated protection of *S. aureus* from antibiotics.

Microbes do not exist in isolation, but rather, as members of a polymicrobial community—a fact that is still underappreciated. We have shown that the antibiotic sensitivity of one microbe can change dramatically and unexpectedly when in the presence of another microbial species, underscoring the difficulty of extrapolating from monoculture experiments to polymicrobial settings. In recent years, other groups have shown other examples of microbial interactions influencing antibiotic effectiveness (17, 61–63). Furthermore, the biofilm mode of growth contributes high-level tolerance to antimicrobial agents and must be considered when studying infections involving biofilms. Clearly, neighboring microbes in a mixed infection can impact this antimicrobial tolerance. Elucidation of the molecular mechanisms and consequences of these interspecies interactions may allow us to better anticipate the outcomes of treating a specific patient’s polymicrobial community with a specific antibiotic. We believe our findings are especially relevant to CF—a chronic, polymicrobial disease that requires continuous treatment with numerous antimicrobial agents.

## MATERIALS AND METHODS

See Text S1 in the supplemental material for additional details regarding the methods.

10.1128/mBio.00873-17.1 TEXT S1  Supplemental materials and methods. Download TEXT S1, PDF file, 0.2 MB. Copyright © 2017 Orazi and O’Toole. 2017 Orazi and O’Toole This content is distributed under the terms of the Creative Commons Attribution 4.0 International license.

### Bacterial strains and culture conditions.

A list of all *S. aureus* and *P. aeruginosa* strains used in this study is included in Table S2 in the supplemental material. *S. aureus* was grown in tryptic soy broth (TSB), and *P. aeruginosa* was grown in lysogeny broth (LB). All overnight cultures were grown with shaking at 37°C for 12 to 14 h, except for the *S. aureus* Col *hemB* mutant, which was grown statically at 37°C for 20 h.

10.1128/mBio.00873-17.10 TABLE S2  Strains used in this study. Download TABLE S2, PDF file, 0.1 MB. Copyright © 2017 Orazi and O’Toole. 2017 Orazi and O’Toole This content is distributed under the terms of the Creative Commons Attribution 4.0 International license.

### Biofilm disruption assay on plastic.

Overnight liquid cultures of *S. aureus* were diluted to an optical density at 600 nm (OD_600_) of 0.05, washed in phosphate-buffered saline (PBS), and resuspended in minimal essential medium (MEM [Thermo, Fisher Scientific]) supplemented with 2 mM l-glutamine (MEM+l-Gln). Triplicate wells of a plastic 96-well plate were inoculated with 100 μl of the *S. aureus* suspensions and incubated at 37°C in 5% CO_2_. Unattached cells were removed 1 h postinoculation (p.i.), and 90 μl of MEM+l-Gln was added to each well. The plate was incubated at 37°C in 5% CO_2_. Unattached cells were removed 6 h p.i., at which point antibiotic dilutions in MEM+l-Gln, *P. aeruginosa* supernatant, and MEM+l-Gln were added (total well volume of 90 μl). The plate was incubated at 37°C in 5% CO_2_. The planktonic cell population was collected 30 h p.i., serially diluted 10-fold in PBS, and plated on mannitol salt agar. After incubation at 37°C for 18 h, planktonic CFU were determined. To collect the remaining biofilm cell population from the 96-well plate, 50 μl of 0.1% Triton X-100 in PBS was added to each well. Next, the plate was gently agitated on an undulating rocker for 30 min, and wells were scraped using a solid multipin replicator. Biofilms were further disrupted by covering the plate with a foil seal and vortexing for 2 min. Biofilm cells were serially diluted, plated, and enumerated as described for the planktonic cells. Viable cell counts for all relevant experiments are reported as log_10_ transformed CFU per milliliter. For time course assays, samples were collected and analyzed 0, 1, 3, 6, 19, and 26 h after the 6-h p.i. medium change.

### MBC assay to test inactivation of vancomycin by *P. aeruginosa* supernatant.

Vancomycin dilutions were prepared in either MEM+l-Gln or *P. aeruginosa* supernatant and incubated for 24 h. Minimum bactericidal concentrations (MBCs) of vancomycin (preincubated with either MEM+l-Gln or *P. aeruginosa* supernatant) for *S. aureus* and *Streptococcus sanguinis* were determined. See Text S1 in the supplemental material for additional details regarding the methods.

### Biofilm disruption assay on CFBE cells.

Overnight liquid cultures of *S. aureus* were diluted to an OD_600_ of 0.05, washed in PBS, and resuspended in MEM+l-Gln. CFBE cells were grown in a plastic 24-well plate until confluent, at which point the CFBE monolayers were washed twice with 500 μl MEM+l-Gln. Next, triplicate wells were inoculated with 500 μl of the *S. aureus* suspensions and incubated at 37°C in 5% CO_2_. Unattached cells were removed 1 h p.i., and 450 μl of MEM+l-Gln was added to each well. The plate was incubated at 37°C, 5% CO_2_. Unattached cells were removed 6 h p.i., at which point 50 μg/ml vancomycin in MEM+l-Gln, *P. aeruginosa* supernatant, and MEM+l-Gln were added (total well volume of 500 μl). Planktonic cell populations were removed 21 h p.i. Then biofilms were disrupted by adding 250 μl of PBS to each well and scraping thoroughly with a plastic pipette tip. Biofilm cells were serially diluted and plated as previously described for the biofilm disruption assay on plastic.

### Bright-field and fluorescence microscopy.

CFBE cells were inoculated with *S. aureus* and *P. aeruginosa*. Microcolonies were imaged at 6 and 21 h p.i. by bright-field and fluorescence microscopy. See Text S1 in the supplemental material for additional details regarding the methods.

### MBC_90_ assay on CFBE cells.

MBC_90_ of vancomycin was determined for planktonic and biofilm populations of *S. aureus* grown on CFBE cells. See Text S1 in the supplemental material for additional details regarding the methods.

### Cytotoxicity assay.

Biofilm disruption assays were performed on CFBE cells as previously described. Supernatants were collected 21 h p.i., and lactate dehydrogenase (LDH) release from the CFBE cells was measured. See Text S1 in the supplemental material for additional details regarding the methods.

### Quantification of *P. aeruginosa* exoproducts.

*P. aeruginosa* was grown either on plastic or on CFBE cells (in MEM+l-Gln) as described previously, and supernatants were collected 6, 21, or 24 h p.i. HQNO levels in supernatants were measured using a standard curve relating pure HQNO concentration to *S. aureus* CFU upon coculture with the *P. aeruginosa* Δ*pqsL* mutant. Pyoverdine and pyocyanin were quantified as previously described (32, 64). See Text S1 in the supplemental material for additional details regarding the methods.

### Other growth assays.

For growth curves in shaking flasks, *S. aureus* was exposed to either HQNO, *P. aeruginosa* supernatant, or MEM+l-Gln alone. Samples were collected every 2 h, and planktonic CFU were determined. For the planktonic susceptibility assay in shaking flasks, *S. aureus* was exposed to either *P. aeruginosa* supernatant or MEM+l-Gln, with or without vancomycin. For the SCV selection assay, *S. aureus* was exposed to either *P. aeruginosa* supernatant or MEM+l-Gln alone. SCV selection was performed as previously described (15). See Text S1 in the supplemental material for additional details regarding the methods.

### Transmission electron microscopy.

*S. aureus* was exposed to *P. aeruginosa* supernatant, HQNO, or MEM+l-Gln alone. Biofilm cells were fixed, stained, and sectioned. Samples were imaged with a transmission electron microscope, and cell wall thickness was measured. See Text S1 in the supplemental material for additional details regarding the methods.

### Biofilm antibiotic susceptibility assay on plastic.

A modified biofilm antibiotic susceptibility assay was performed using Biolog Phenotype MicroArray bacterial chemical sensitivity panels. See Text S1 in the supplemental material for additional details regarding the methods.
