# Supplementary figures and images for: Pseudomonas aeruginosa Alters Staphylococcus aureus Sensitivity to Vancomycin in a Biofilm Model of Cystic Fibrosis Infection
Source: mBio. 2017 Jul 18;8(4):e00873-17. doi: 10.1128/mBio.00873-17 (PMC5516255; doi:10.1128/mBio.00873-17)

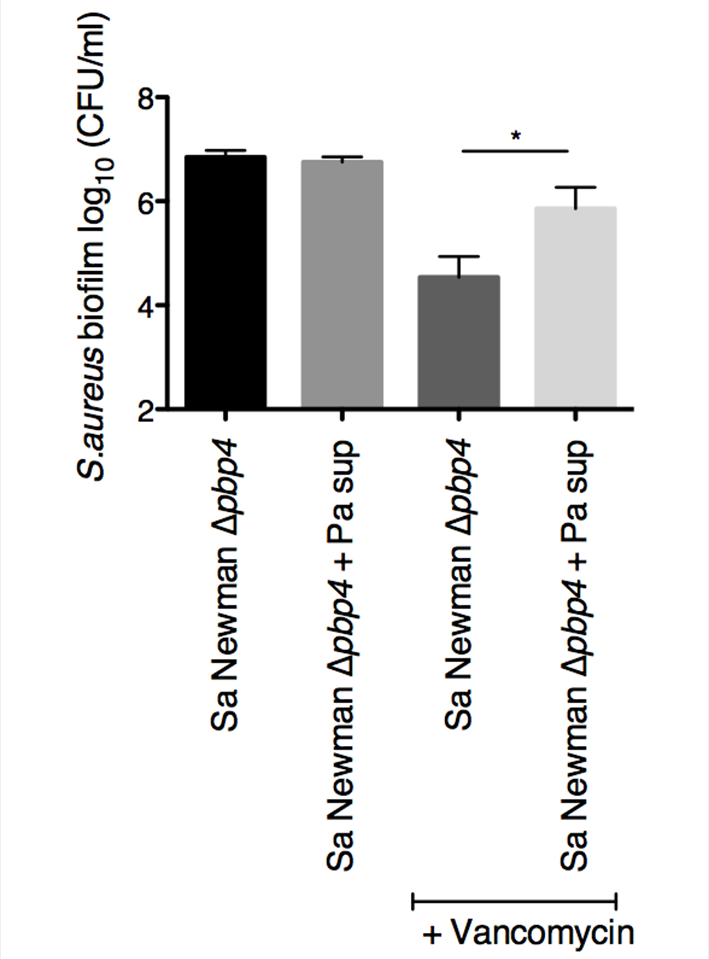

Supplement: FIG S1 [file mbo004173384sf1.tif]

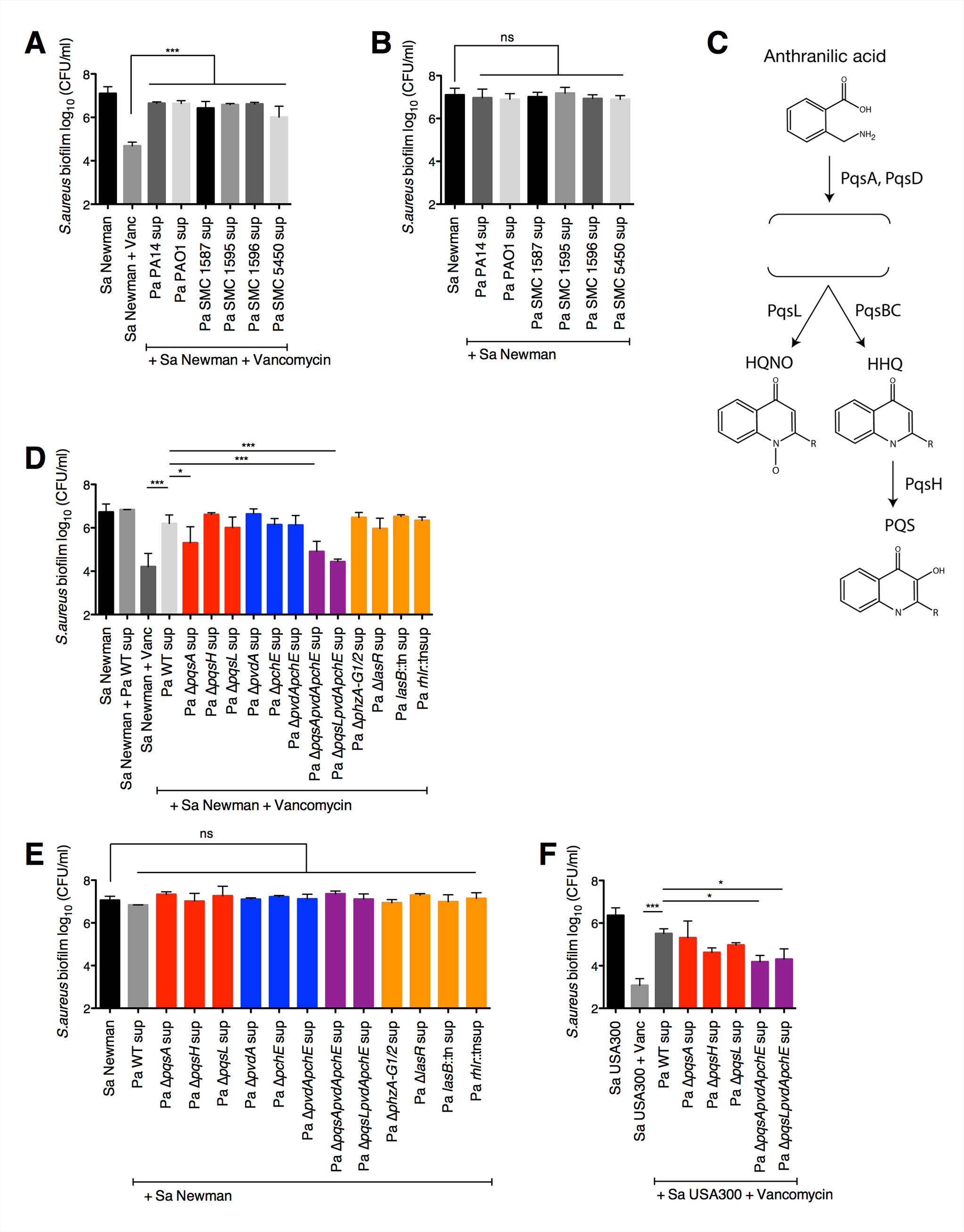

Supplement: FIG S2 [file mbo004173384sf2.tif]

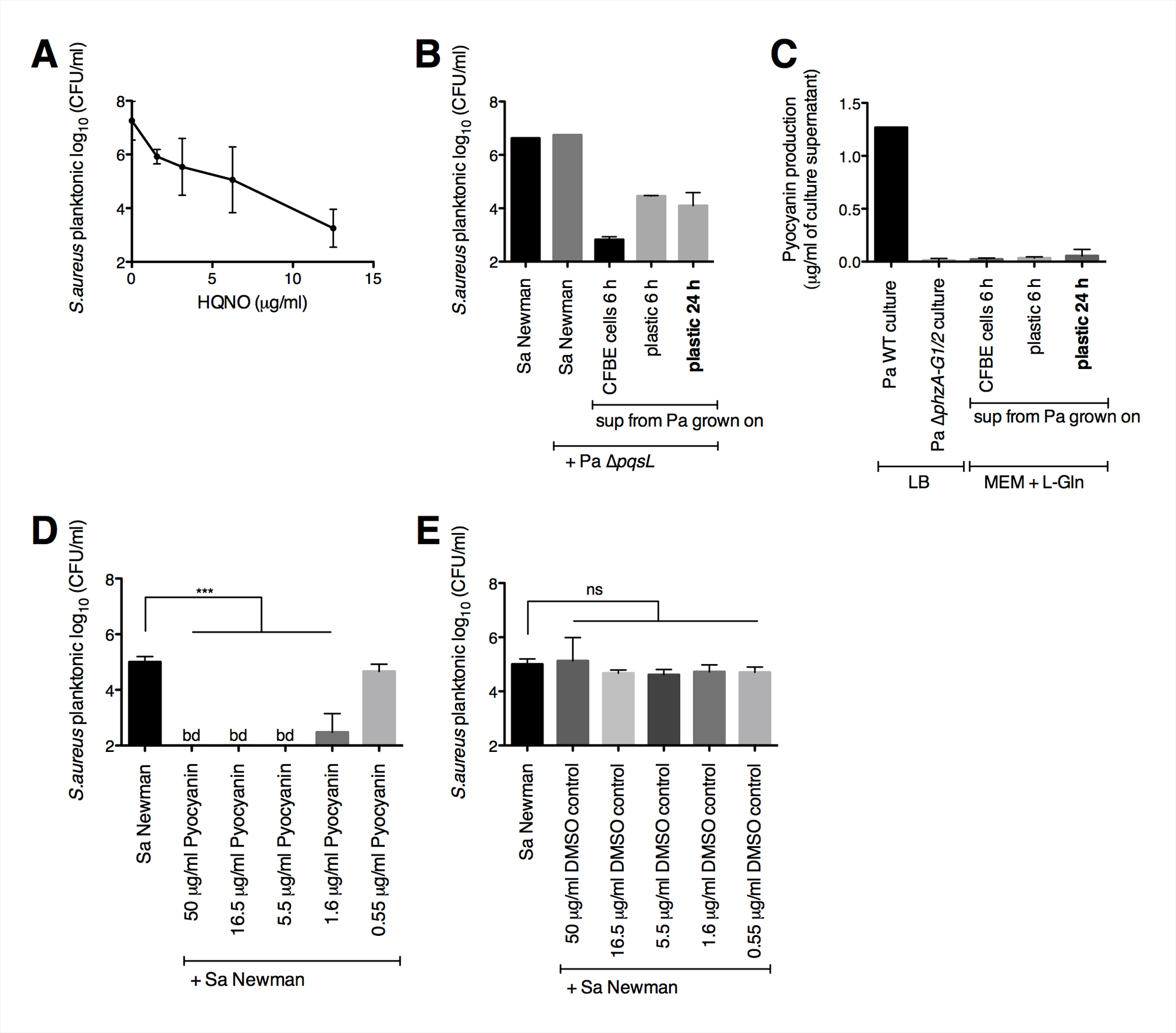

Supplement: FIG S3 [file mbo004173384sf3.tif]

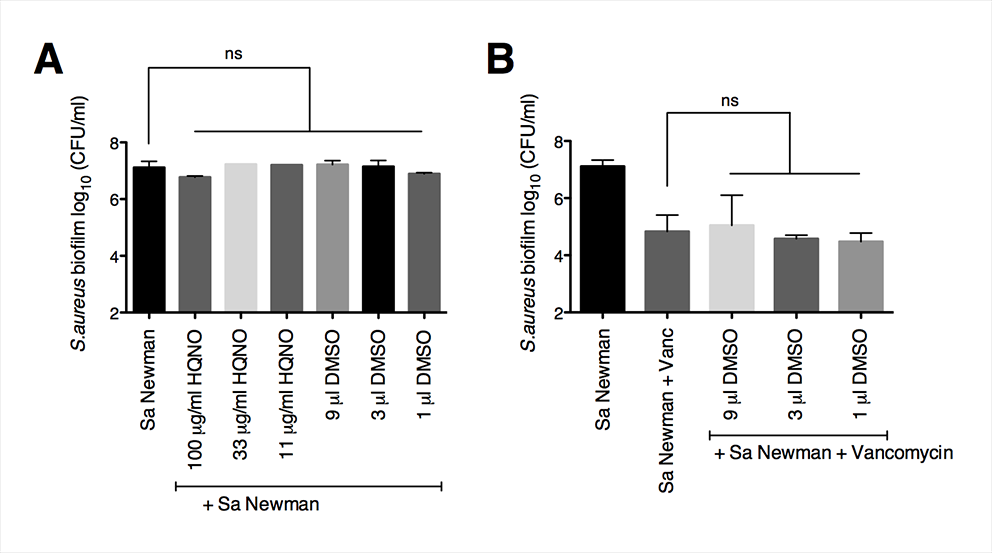

Supplement: FIG S4 [file mbo004173384sf4.tif]

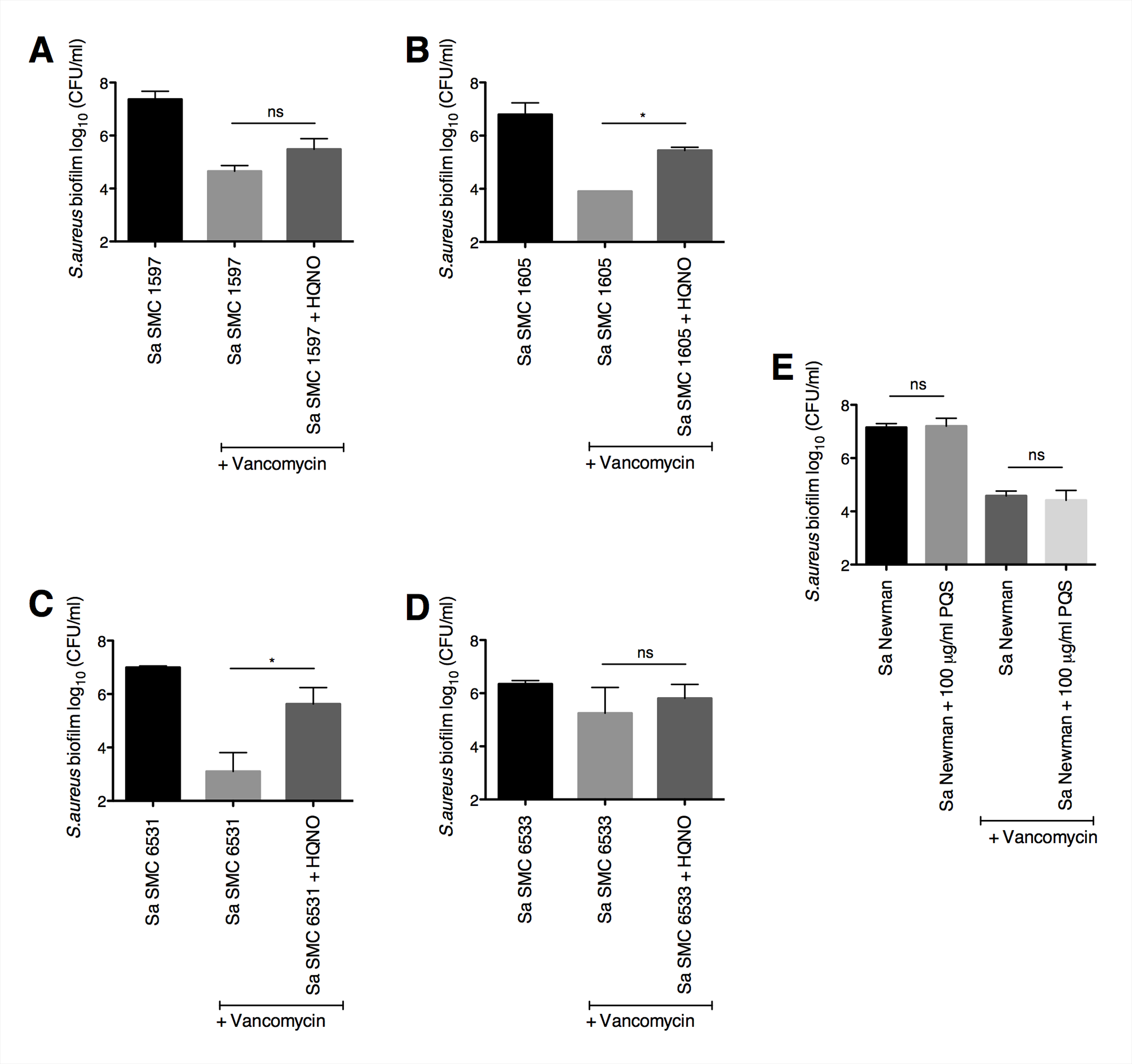

Supplement: FIG S5 [file mbo004173384sf5.tif]

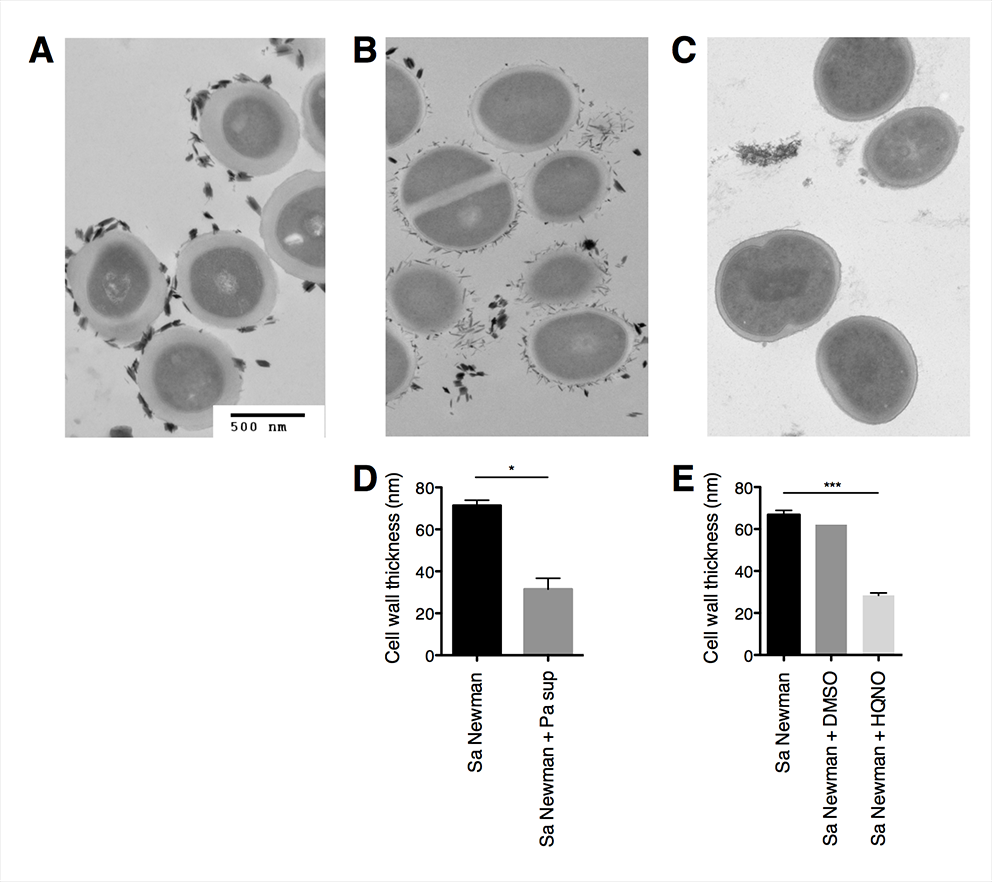

Supplement: FIG S6 [file mbo004173384sf6.tif]

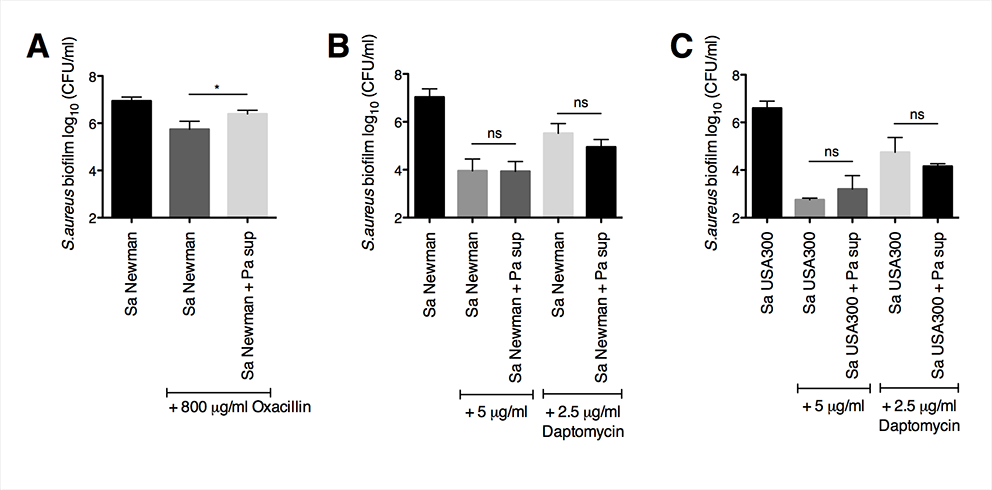

Supplement: FIG S7 [file mbo004173384sf7.tif]
