# Supplementary material for: Pseudomonas aeruginosa Alters Staphylococcus aureus Sensitivity to Vancomycin in a Biofilm Model of Cystic Fibrosis Infection
Source: mBio. 2017 Jul 18;8(4):e00873-17. doi: 10.1128/mBio.00873-17 (PMC5516255; doi:10.1128/mBio.00873-17)
Supplement: TABLE S1 [file mbo004173384st1.pdf]

Supplemental Table 1. ***P. aeruginosa*** **supernatant protects** ***S. aureus*** **from several classes of antibiotics.**

Mechanism of action	Class	Name	Population protected[a]	Protection requires HQNO and siderophores
Cell wall synthesis inhibition	β-lactam	Amoxicillin	Both	-
		Azlocillin	Both	-
		Carbenicillin	Both	Yes
		Cefazolin	Both	-
		Cefmetazole	Both	-
		Cefsulodin	Biofilm only	-
		Cefuroxime	Biofilm only	-
		Cephalothin	Both	-
		Cloxacillin	Both	-
		Nafcillin	Both	-
		Oxacillin	Biofilm only	-
		Penicillin G	Both	Yes
		Phenethicillin	Planktonic only	-
	Glycopeptide	Phleomycin	Both	-
		Vancomycin	Both	Yes
	Other	D-Cycloserine	Both	-
Protein synthesis inhibition	Aminoglycoside	Neomycin	Planktonic only	-
		Paromomycin	Planktonic only	Yes
		Sisomicin	Planktonic only	Yes
		Streptomycin	Planktonic only	-
		Tobramycin	Planktonic only	Yes
	Aminonucleoside	Puromycin	Both	-
	Lincosamide	Lincomycin	Both	-
	Macrolide	Erythromycin	Planktonic only	-
		Josamycin	Planktonic only	-
		Oleandomycin	Planktonic only	-
		Spiramycin	Planktonic only	Yes
		Tylosin	Planktonic only	-
	Polypeptide	Capreomycin	Planktonic only	-
	Steroid	Fusidic acid	Planktonic only	-
	Tetracycline	Chlortetracycline	Planktonic only	-

		Doxycycline	Planktonic only	-
		Minocycline	Both	-
		Penimepicycline	Planktonic only	Yes
		Rolitetracycline	Planktonic only	-
Nucleic acid inhibition	Rifamycin	Rifampicin	Biofilm only	Yes
	Aminocoumarin	Novobiocin	Both	Yes
Other	Quaternary ammonium cation	Benzethonium chloride	Biofilm only	-
		Cetylpyridinium chloride	Both	-
		Dequalinium chloride	Biofilm only	-
		Dodecyltrimethyl ammonium bromide	Both	-
		Sanguinarine	Both	-
	Sodium compound	Sodium arsenate	Both	-
		Sodium metaborate	Planktonic only	-
		Sodium metavanadate	Planktonic only	-
		Sodium tungstate	Planktonic only	-
	Other	1,10-Phenanthroline	Planktonic only	-
		18-Crown-6-ether	Planktonic only	-
		5-fluoro-5'-deoxyuridine	Both	-
		5-Fluorouracil	Both	-
		Beta-Chloro-L-alanine hydrochloride	Both	-
		Boric acid	Planktonic only	-
		Chelerythrine	Biofilm only	-
		Crystal violet	Both	-
		D-Serine	Planktonic only	-
		D,L-Thioctic acid	Planktonic only	-
		Dichloro-8-hydroxyquinaldine	Planktonic only	-
		Glycine hydroxamate	Planktonic only	-

Guanidine hydrochloride	Both	-
Hydroxyquinoline	Planktonic only	-
Lawsone	Both	-
Manganese chloride	Both	-
Methyl viologen	Both	-
Myricetin	Planktonic only	-
Niaproof	Planktonic only	-
Patulin	Both	-
Poly-L-lysine	Both	-
Protamine sulfate	Both	-
Tannic acid	Both	-
Tetrazolium violet	Biofilm only	-
Thioridazine	Biofilm only	-

-; denotes antibiotics that were not tested with *P. aeruginosa* PA14 Δ*pqsLpvdApchE* mutant supernatant.

[a]Population protected; Denotes whether biofilm, planktonic, or both populations were protected by wild-type *P. aeruginosa* PA14 supernatant. Protection is defined as at least a 10-fold increase in CFUs between *S. aureus* exposed to *P. aeruginosa* supernatant (in addition to antibiotic) and *S. aureus* exposed to antibiotic alone.
