# Supplementary material for: Pseudomonas aeruginosa Alters Staphylococcus aureus Sensitivity to Vancomycin in a Biofilm Model of Cystic Fibrosis Infection
Source: mBio. 2017 Jul 18;8(4):e00873-17. doi: 10.1128/mBio.00873-17 (PMC5516255; doi:10.1128/mBio.00873-17)
Supplement: TEXT S1 [file mbo004173384s1.pdf]

Supplemental Materials and Methods

Preparation of *P. aeruginosa* supernatants. Overnight liquid cultures of *P. aeruginosa* were diluted to an OD_{600} of 0.05, washed in phosphate-buffered saline (PBS), and resuspended in minimal essential medium (MEM, ThermoFisher Scientific) supplemented with 2 mM L-glutamine (MEM + L-Gln). Next, each well of a plastic 6-well plate was inoculated with 2 ml of the *P. aeruginosa* suspensions and incubated at 37°C, 5% CO_2 for 1 h. Subsequently, unattached cells were removed and 2 ml of MEM + L-Gln was added to each well. Following incubation for an additional 22-24 h at 37°C, 5% CO_2, the culture supernatant were collected, centrifuged at 5,000 x *g* for 5 min, and passed through a 0.22-μm filter. In subsequent experiments, wells containing *P. aeruginosa* supernatant received half the well volume of supernatant and half the well volume of either MEM + L-Gln or antibiotic solutions in MEM + L-Gln.

MBC assay to test inactivation of vancomycin by *P. aeruginosa* supernatant. Two-fold dilutions of vancomycin (ranging from 500 μg/ml to 0.49 μg/ml) were prepared in either MEM + L-Gln or *P. aeruginosa* supernatant. *P. aeruginosa* supernatant was prepared from *P. aeruginosa* cells grown in MEM + L-Gln for 24 hrs as described in the main text. The following were added to a sterile 96-well plate in duplicate wells: 50 μl of MEM + L-Gln, 50 μl of vancomycin dilutions in MEM + L-Gln, 50 μl of *P. aeruginosa* supernatant, and 50 μl of vancomycin dilutions in *P. aeruginosa* supernatant. The plate was incubated at room temperature for 24 h. Overnight liquid cultures of *S. aureus* Newman and *Streptococcus sanguinis* were diluted to an OD_{600} of 0.05, washed in PBS, and resuspended in MEM + L-Gln. Then, 50 μl of the bacterial suspension added to each condition, resulting in a total well volume of 100 μl. Following incubation for an additional 24 h at 37°C, 5% CO_2, a solid multi-pin replicator was used to

scrape the bottom of the wells and transfer ~2 µl onto either TSA or blood agar to grow *S. aureus* and *S. sanguinis*, respectively. Blood agar plates were placed in a Gas-pak container. After incubation at 37°C for 18 h, minimum bactericidal concentrations (MBCs) of vancomycin pre-incubated with MEM + L-Gln and with *P. aeruginosa* supernatant were determined for *S. aureus* and *S. sanguinis* by observing presence or absence of growth on the agar plates.

Brightfield and fluorescence microscopy. CFBE cells were grown in 35 mm glass bottom dishes (Cellvis) until confluent, at which point the CFBE cell monolayers were washed twice in MEM + L-Gln. *S. aureus* RN6390, or *S. aureus* RN6390 labeled with DsRed and *P. aeruginosa* PAO1 labeled with GFP were used in this experiment. Overnight liquid cultures of *S. aureus* and *P. aeruginosa* were diluted to an OD_{600} of 0.025, washed in PBS, and resuspended in MEM + L-Gln. Dishes were inoculated with either 1 ml of *S. aureus* suspension and 1 ml *P. aeruginosa* suspension, or 1 ml of *S. aureus* suspension and 1 ml MEM + L-Gln, and incubated at 37°C, 5% CO_2. One hour post-inoculation, unattached cells were removed and 2 ml MEM + L-Gln was added to each dish, followed by incubation at 37°C, 5% CO_2. At either 6 or 21 h p.i., unattached cells were removed and the dishes were visualized using the Nikon Eclipse Ti inverted microscope using the 100x objective. Images were taken using Nikon ND acquisition software and a Hamamatsu C11440 Orca-Flash 4.0 digital camera.

MBC_{90} assay on CFBE cells. Overnight liquid cultures of *S. aureus* were diluted to an OD_{600} of 0.05, washed in PBS, and resuspended in MEM + L-Gln. CFBE cells were grown in a plastic 24-well plate until confluent, at which point the CFBE monolayers were washed twice with 500 µl MEM + L-Gln. Next, duplicate wells were inoculated with 500 µl of the *S. aureus* suspensions

and incubated at 37°C, 5% CO_2. Unattached cells were removed 1 h p.i., and 450 μl of MEM + L-Gln was added to each well. The plate was incubated at 37°C, 5% CO_2. Unattached cells were removed 6 h p.i., at which point two-fold dilutions of vancomycin (ranging from 500 μg/ml to 0.49 μg/ml) MEM + L-Gln were added (total well volume of 500 μl). Planktonic cell populations were removed 21 h p.i., serially diluted, and plated as previously described. Then, biofilms were disrupted by adding 250 μl of PBS to each well and scraping thoroughly with a plastic pipette tip. Biofilm cells were serially diluted and plated as previously described for the biofilm disruption assay on plastic. Planktonic and biofilm CFUs were counted and MBC_{90} (the lowest antibiotic concentration that kills 90% of the population) was determined for both fractions.

Cytotoxicity assay. Biofilm disruption assays were performed on CFBE cells as previously described. Unattached cells were removed 6 h p.i., and then the following treatments were added in triplicate: *P. aeruginosa* PA14 wild-type or Δ*pqsLpvdApchE* deletion mutant supernatant (either 1x or 1/16x), 50 μg/ml vancomycin in MEM + L-Gln, and MEM + L-Gln. Supernatants were collected 21 h p.i. and lactate dehydrogenase (LDH) release from the CFBE cells was measured using the Cyto Tox 96 Non-Radioactive Cytotoxicity Assay (Promega) according to the manufacturer's instructions. Cytotoxicity was measured as the fraction of LDH release compared to total LDH release by CFBE cells treated with 0.1% Triton X-100 in PBS to completely lyse cells.

HQNO quantification. A co-culture assay was performed with the *P. aeruginosa* Δ*pqsL* mutant, *S. aureus*, and dilutions of pure HQNO to create a standard curve relating pure HQNO concentration to *S. aureus* CFUs. The *P. aeruginosa* Δ*pqsL* mutant does not produce HQNO, and

does not kill *S. aureus*; killing can be restored by adding exogenous HQNO. Overnight liquid cultures of the *P. aeruginosa* Δ*pqsL* mutant and *S. aureus* were diluted to an OD_{600} of 0.1, washed in PBS, and resuspended in TSB. Triplicate wells of a plastic 96-well plate were inoculated with the *P. aeruginosa* Δ*pqsL* mutant and *S. aureus* suspensions, and dilutions of pure HQNO in MEM + L-Gln (12.5 μg/ml, 6.25 μg/ml, 3.125 μg/ml, 1.56 μg/ml; total well volume of 100 μl), and incubated at 37°C, 5% CO_2. Planktonic cell populations were collected 26 h p.i., serially diluted, and plated as previously described. A standard curve was constructed to relate pure HQNO concentration to *S. aureus* planktonic CFUs.

P. aeruginosa was grown either on plastic or on CFBE cells (in MEM + L-Gln) as described previously, and supernatants were collected 6 h or 24 h p.i. A co-culture assay was performed as follows: overnight liquid cultures of the *P. aeruginosa* Δ*pqsL* mutant and *S. aureus* were diluted to an OD_{600} of 0.1, washed in PBS, and resuspended in TSB. Triplicate wells of a plastic 96-well plate were inoculated with the *P. aeruginosa* Δ*pqsL* mutant and *S. aureus* suspensions, and supernatants from *P. aeruginosa* grown either on plastic or on CFBE cells (total well volume of 100 μl), and incubated at 37°C, 5% CO_2. Planktonic cell populations were collected 26 h p.i., serially diluted, and plated as previously described. HQNO levels in supernatants from *P. aeruginosa* grown either on plastic or on CFBE cells were determined using the standard curve constructed above.

Pyoverdine quantification. *P. aeruginosa* was grown either on plastic or on CFBE cells (in MEM + L-Gln) as described previously and supernatants were collected at 6 h, 21 h, or 24 h p.i.

Cells were centrifuged at 10,000 x *g* for 2 min and supernatants were collected. Absorbance at 405 nm was measured, as previously reported (1).

Pyocyanin quantification. *P. aeruginosa* was grown either on plastic or on CFBE cells (in MEM + L-Gln) as described above and supernatants were collected at 6 h or 24 h p.i. Supernatants were also collected from overnight liquid cultures of *P. aeruginosa* wild-type or Δ*phzA-G1/2* grown in LB as controls. Cells were centrifuged at 10,000 x *g* for 2 min and supernatants were collected. Supernatants were extracted with chloroform and then with 0.2 N HCl, and absorbance at 520 nm was measured, as previously reported (2). Absorbance values were multiplied by the extinction coefficient 17.052 to determine the concentration of pyocyanin per ml of supernatant.

Biofilm disruption assay on plastic with pyocyanin. Pure pure pyocyanin (Santa Cruz Biotechnology) was dissolved in DMSO and diluted in MEM + L-Gln. Triplicate wells of a plastic 96-well plate were inoculated with *S. aureus* as described for the biofilm disruption assay on plastic and incubated at 37°C, 5% CO_2. Unattached cells were removed 1 h p.i., and 90 µl of MEM + L-Gln was added to each well. The plate was incubated at 37°C, 5% CO_2. Unattached cells were removed 6 h p.i., at which point pyocyanin dilutions in MEM + L-Gln (50 µg/ml, 16.5 µg/ml, 5.5 µg/ml, 1.6 µg/ml, 0.55 µg/ml) and MEM + L-Gln were added to the appropriate wells (total well volume of 90 µl) and incubated at 37°C, 5% CO_2. Planktonic cell populations were collected 30 h p.i., serially diluted, and plated as previously described.

Biofilm disruption assay on plastic with 2-n-Heptyl-4-hydroxyquinoline N-oxide. Pure 2-n-

Heptyl-4-hydroxyquinoline N-oxide (HQNO, Santa Cruz Biotechnology) was dissolved in DMSO and diluted in MEM + L-Gln. Triplicate wells of a plastic 96-well plate were inoculated with *S. aureus* as described for the biofilm disruption assay on plastic and incubated at 37°C, 5% CO_2. Unattached cells were removed 1 h p.i., and 90 μl of MEM + L-Gln was added to each well. The plate was incubated at 37°C, 5% CO_2. Unattached cells were removed 6 h p.i., at which point HQNO dilutions in MEM + L-Gln (100 μg/ml, 33 μg/ml, 11 μg/ml), 50 μg/ml vancomycin in MEM + L-Gln, and MEM + L-Gln were added to the appropriate wells (total well volume of 90 μl) and incubated at 37°C, 5% CO_2. Planktonic cell populations were removed 30 h p.i. Biofilms were disrupted, serially diluted, and plated as previously described for the biofilm disruption assay on plastic.

Biofilm disruption assay on plastic under anoxic conditions. Oxygen-depleted MEM + L-Gln was prepared by incubating a conical tube of medium at 1% oxygen overnight. Triplicate wells of two plastic 96-well plates (one exposed to normoxia, one to anoxia) were inoculated with *S. aureus* as described for the biofilm disruption assay on plastic. The plates were incubated at 37°C, 5% CO_2. Unattached cells were removed 1 h p.i., and 90 μl of MEM + L-Gln (either regular or oxygen-depleted) was added to each well and incubated at 37°C, 5% CO_2. Unattached cells were removed 6 h p.i., at which point 50 μg/ml vancomycin in MEM + L-Gln (either regular or oxygen-depleted), *P. aeruginosa* PA14 supernatant (either regular or oxygen-depleted), and MEM + L-Gln (either regular or oxygen-depleted) were added to the appropriate wells (total well volume of 90 μl). One plate was incubated at 37°C, 5% CO_2, while the other plate was placed in a Gas-pak container at 37°C. Planktonic cell populations were removed 30 h

p.i. Biofilms were disrupted, serially diluted, and plated as previously described for the biofilm disruption assay on plastic.

Growth curves in shaking flasks. Overnight liquid cultures of *S. aureus* Newman were diluted to an OD_{600} of 0.05, washed in PBS, and resuspended in either MEM + L-Gln, 100 µg/ml HQNO in MEM + L-Gln, or *P. aeruginosa* PA14 wild-type or Δ*pqsLpvdApchE* deletion mutant supernatant (total volume of 50 ml). Duplicate 125 ml flasks were inoculated with the *S. aureus* suspensions and incubated at 37°C, shaking at 230 rpm. Samples were collected every 2 h from 0 to 10 h p.i., and from 12 to 24 h p.i. Planktonic CFUs were determined as previously described for the biofilm disruption assay on plastic.

Planktonic susceptibility assay in shaking flasks. Overnight liquid cultures of *S. aureus* Newman were diluted to an OD_{600} of 0.05, washed in PBS, and resuspended in either MEM + L-Gln, 50 µg/ml vancomycin in MEM + L-Gln, or *P. aeruginosa* PA14 supernatant. Duplicate flasks were inoculated with the *S. aureus* suspensions and incubated at 37°C, shaking at 230 rpm. Planktonic CFUs were determined 24 h p.i. as previously described for the biofilm disruption assay on plastic.

Small colony variant selection assay. Triplicate wells of a plastic 96-well plate were inoculated with *S. aureus* Newman as described for the biofilm disruption assay on plastic. Unattached cells were removed 1 h p.i., and 90 µl of MEM + L-Gln was added to each well. Unattached cells were removed 6 h p.i. Subsequently, *P. aeruginosa* PA14 wild-type supernatant, Δ*pqsLpvdApchE* deletion mutant supernatant, and MEM + L-Gln were added to appropriate

wells (total well volume of 90 μl). After incubation at 37°C, 5% CO_2 for either 1 or 5 additional days, planktonic cell populations were removed from the plates and biofilms were disrupted and serially diluted as previously described for the biofilm disruption assay on plastic. A small colony variant (SCV) selection assay was performed as previously described (3). Serial dilutions were spread onto tryptic soy agar plates with or without 4 μg/ml gentamicin. After incubation at 37°C for 5 days, plates were examined for the appearance of SVCs. SCVs were defined as colonies that are 10x smaller than normal *S. aureus* colonies (4, 5). This experiment was repeated three times.

Transmission electron microscopy. An overnight liquid culture of *S. aureus* Newman was diluted to an OD_{600} of 0.1, washed in PBS, and resuspended in MEM + L-Gln. Each well of plastic 6-well plates were inoculated with 2 ml of the *S. aureus* suspension and then incubated at 37°C, 5% CO_2. Unattached cells were removed 1 h p.i., at which point either 1 ml of *P. aeruginosa* supernatant plus 1 ml of MEM + L-Gln, 2 ml of 100 μg/ml HQNO in MEM + L-Gln, or 2 ml of MEM + L-Gln alone (control) was added to the appropriate wells (total well volume of 2 ml). Planktonic cell populations were removed from the plates 25 h p.i. Afterwards, a 10X volume of 2% glutaraldehyde-tannic acid/1% paraformaldehyde in 0.1 M sodium cacodylate buffer (pH 7.2-7.4) was added to each well to fix the cells and cell scrapers were used to detach the biofilm cells. Next, cells were centrifuged at 600 x *g* for 10 min. Then, the samples were post-fixed in 1% OsO4 in sodium cacodylate buffer (pH 7.4), stained en-bloc using 1-2% uranyl acetate, and dehydrated using increasing concentrations of EtOH. Pellets were immersed in LX112 resin:propylene oxide, dessicated, and heated. Once samples were sectioned, images were taken using a JEOL JEM 1010 transmission electron microscope at 100KV and 5,000X

magnification. Cell wall thickness was measured for cells with nearly equatorial-cut surfaces. For each technical replicate, at least 50 cells were measured.

Biofilm antibiotic susceptibility assay on plastic. To assay biofilm and planktonic antibiotic susceptibility versus a range of antimicrobial agents, we utilized the Phenotype MicroArray bacterial chemical sensitivity assay panels 12 – 20 from Biolog. Overnight liquid cultures of *S. aureus* Newman were diluted to an OD_{600} of 0.05, washed in PBS, and resuspended in MEM + L-Gln. For each Phenotype MicroArray panel, half-area 96-well plastic plates were inoculated with 50 μl of *S. aureus* suspension and either 50 μl of MEM + L-Gln or 50 μl of *P. aeruginosa* PA14 wild-type supernatant. The plates were incubated at 37°C, 5% CO_2. The planktonic cell population was collected 24 h p.i., and then serially diluted and plated as previously described for the biofilm disruption assay on plastic. To collect the remaining biofilm cell population from the 96-well plates, 50 μl of 0.1% Triton X-100 in PBS was added to each well. Next, the plates were gently agitated on an undulating rocker for 60 min. Biofilms were further disrupted by covering the plates with a foil seal and vortexing for 2 min. Biofilm cells were serially diluted, plated, and enumerated as described for the planktonic cells. This assay was repeated for Phenotype MicroArray Panel 12 with the inclusion of *P. aeruginosa* PA14 Δ*pqsLpvdApchE* deletion mutant supernatant.

Literature Cited

1. **Hohnadel D**, **Haas D**, **Meyer J-M**. 1986. Mapping of mutations affecting pyoverdine production in *Pseudomonas aeruginosa*. FEMS Microbiol Lett **36**:195–199.

2. **Essar DW**, **Eberly L**, **Hadero A**, **Crawford IP**. 1990. Identification and characterization of genes for a second anthranilate synthase in *Pseudomonas aeruginosa*: interchangeability of the two anthranilate synthases and evolutionary implications. J Bacteriol **172**:884–900.

3. **Mitchell G**, **Séguin DL**, **Asselin A-E**, **Deziel E**, **Cantin AM**, **Frost EH**, **Michaud S**, **Malouin F**. 2010. *Staphylococcus aureus* sigma B-dependent emergence of small-colony variants and biofilm production following exposure to *Pseudomonas aeruginosa* 4-hydroxy-2-heptylquinoline-N-oxide. BMC Microbiol **10**:33.

4. **Hoffman LR**, **Deziel E**, **Lepine F**, **Emerson J**, **McNamara S**, **Gibson RL**, **Ramsey BW**, **Miller SI**. 2006. Selection for *Staphylococcus aureus* small-colony variants due to growth in the presence of *Pseudomonas aeruginosa*. Proceedings of the National Academy of Sciences **103**:19890–19895.

5. **Proctor RA**, **Eiff von C**, **Kahl BC**, **Becker K**, **McNamara P**, **Herrmann M**, **Peters G**. 2006. Small colony variants: a pathogenic form of bacteria that facilitates persistent and recurrent infections. Nat Rev Micro **4**:295–305.
