# Supplementary material for: Pseudomonas aeruginosa Alters Staphylococcus aureus Sensitivity to Vancomycin in a Biofilm Model of Cystic Fibrosis Infection
Source: mBio. 2017 Jul 18;8(4):e00873-17. doi: 10.1128/mBio.00873-17 (PMC5516255; doi:10.1128/mBio.00873-17)
Supplement: TABLE S2 [file mbo004173384st2.pdf]

Supplemental Table S2. Strains used in this study.

Species and strain	Strain number	Phenotype	Source / reference
S. aureus			
Newman	SMC 1007	MSSA[a]	(1)
Newman Δ*pbp4*	ALC 4815	MSSA	(2)
USA300	SMC 6971	MRSA[b]	(3)
Col	SMC 6598	MRSA	(4)
Col *hemB* mutant	SMC 6599, ALC 4689	MRSA, SCV[c]	(5)
RN6390/pSK236	SMC 1308	DsRed-expressing	A. L. Cheung
Clinical isolate	SMC 1597	MSSA	(6)
Clinical isolate	SMC 1603	MSSA	(6)
Clinical isolate	SMC 1605	MSSA	(6)
Clinical isolate	SMC 6531	MRSA	A. L. Cheung
Clinical isolate	SMC 6532	MRSA	A. L. Cheung
Clinical isolate	SMC 6533	MRSA	A. L. Cheung
P. aeruginosa			
PA14	SMC 232	non-mucoid	(7)
PAO1	SMC 17	non-mucoid	(8)
PAO1/pSMC21	SMC 886	GFP-expressing	Laboratory collection
PAO1 *rhlA*::Gm	SMC 622		(9)
PA14 Δ*pqsA*	SMC 5013		L. G. Rahme
PA14 Δ*pqsH*	SMC 5017		(10)
PA14 Δ*pqsL*	SMC 6216		(6)
PA14 Δ*pvdA*	SMC 6596		(6)
PA14 Δ*pchE*	SMC 6597		(6)
PA14 Δ*pvdA*Δ*pchE*	SMC 6215		(11)
PA14 Δ*pqsA*Δ*pvdA*Δ*pchE*	SMC 6218		Laboratory collection
PA14 Δ*pqsL*Δ*pvdA*Δ*pchE*	SMC 6219		(6)
PA14 Δ*phzA-G1/2*	SMC 5020		(12)
PA14 Δ*lasR*	SMC 5021		(13)
PA14 *lasB*::tn	TML[d]		(14)
PA14 *rhlRr*::tn	TML		(14)
Clinical isolate	SMC 1587	mucoid	(15)
Clinical isolate	SMC 1595	non-mucoid	(15)
Clinical isolate	SMC 1596	non-mucoid	(15)
Clinical isolate	SMC 5450	mucoid	(15)
S. sanguinis			
SK36	SMC 7474		(16)

[a]MSSA, methicillin-sensitive *S. aureus*
[b]MRSA, methicillin-resistant *S. aureus*
[c]SCV, small colony variant
[d]TML, *P. aeruginosa* PA14 NR transposon mutant library

Literature Cited.

1. **Duthie ES**. 1952. Variation in the antigenic composition of staphylococcal coagulase. J Gen Microbiol **7**:320–326.

2. **Memmi G**, **Filipe SR**, **Pinho MG**, **Fu Z**, **Cheung A**. 2008. *Staphylococcus aureus* PBP4 is essential for beta-lactam resistance in community-acquired methicillin-resistant strains. Antimicrob Agents Chemother **52**:3955–3966.

3. **McDougal LK**, **Steward CD**, **Killgore GE**, **Chaitram JM**, **McAllister SK**, **Tenover FC**. 2003. Pulsed-field gel electrophoresis typing of oxacillin-resistant *Staphylococcus aureus* isolates from the United States: establishing a national database. J Clin Microbiol **41**:5113–5120.

4. **Dyke KG**, **Jevons MP**, **Parker MT**. 1966. Penicillinase production and intrinsic resistance to penicillins in *Staphylococcus aureus*. Lancet **1**:835–838.

5. **Vaudaux P**, **Francois P**, **Bisognano C**, **Kelley WL**, **Lew DP**, **Schrenzel J**, **Proctor RA**, **McNamara PJ**, **Peters G**, **Eiff von C**. 2002. Increased expression of clumping factor and fibronectin-binding proteins by *hemB* mutants of *Staphylococcus aureus* expressing small colony variant phenotypes. Infect Immun **70**:5428–5437.

6. **Filkins LM**, **Graber JA**, **Olson DG**, **Dolben EL**, **Lynd LR**, **Bhuju S**, **O'Toole GA**. 2015. Coculture of *Staphylococcus aureus* with *Pseudomonas aeruginosa* drives *S. aureus* towards fermentative metabolism and reduced viability in a cystic fibrosis model. J Bacteriol **197**:2252–2264.

7. **Rahme LG**, **Stevens EJ**, **Wolfort SF**, **Shao J**, **Tompkins RG**, **Ausubel FM**. 1995. Common virulence factors for bacterial pathogenicity in plants and animals. Science **268**:1899–1902.

8. **Holloway BW**, **Morgan AF**. 1986. Genome organization in *Pseudomonas*. Annu Rev Microbiol **40**:79–105.

9. **Rahim R**, **Ochsner UA**, **Olvera C**, **Graninger M**, **Messner P**, **Lam JS**, **Soberón-Chávez G**. 2001. Cloning and functional characterization of the *Pseudomonas aeruginosa rhlC* gene that encodes rhamnosyltransferase 2, an enzyme responsible for di-rhamnolipid biosynthesis. Mol Microbiol **40**:708–718.

10. **Cugini C**, **Morales DK**, **Hogan DA**. 2010. *Candida albicans*-produced farnesol stimulates *Pseudomonas* quinolone signal production in LasR-defective *Pseudomonas aeruginosa* strains. Microbiology (Reading, Engl) **156**:3096–3107.

11. **Wang Y**, **Wilks JC**, **Danhorn T**, **Ramos I**, **Croal L**, **Newman DK**. 2011. Phenazine-1-carboxylic acid promotes bacterial biofilm development via ferrous iron acquisition. J Bacteriol **193**:3606–3617.

12. **Dietrich LEP**, **Price-Whelan A**, **Petersen A**, **Whiteley M**, **Newman DK**. 2006. The

phenazine pyocyanin is a terminal signalling factor in the quorum sensing network of *Pseudomonas aeruginosa*. Mol Microbiol **61**:1308–1321.

13. **Hogan DA**, **Vik A**, **Kolter R**. 2004. A *Pseudomonas aeruginosa* quorum-sensing molecule influences *Candida albicans* morphology. Mol Microbiol **54**:1212–1223.

14. **Liberati NT**, **Urbach JM**, **Miyata S**, **Lee DG**, **Drenkard E**, **Wu G**, **Villanueva J**, **Wei T**, **Ausubel FM**. 2006. An ordered, nonredundant library of *Pseudomonas aeruginosa* strain PA14 transposon insertion mutants. Proceedings of the National Academy of Sciences **103**:2833–2838.

15. **Yu Q**, **Griffin EF**, **Moreau-Marquis S**, **Schwartzman JD**, **Stanton BA**, **O'Toole GA**. 2012. In vitro evaluation of tobramycin and aztreonam versus *Pseudomonas aeruginosa* biofilms on cystic fibrosis-derived human airway epithelial cells. J Antimicrob Chemother **67**:2673–2681.

16. **Hsu SD**, **Cisar JO**, **Sandberg AL**, **Kilian M**. 1994. Adhesive properties of viridans *Streptoccocal* species. Microbial Ecology in Health & Disease **7**:125-137.
